# Epigenetic and transcriptional determinants of the human breast

**DOI:** 10.1038/ncomms7351

**Published:** 2015-02-18

**Authors:** Philippe Gascard, Misha Bilenky, Mahvash Sigaroudinia, Jianxin Zhao, Luolan Li, Annaick Carles, Allen Delaney, Angela Tam, Baljit Kamoh, Stephanie Cho, Malachi Griffith, Andy Chu, Gordon Robertson, Dorothy Cheung, Irene Li, Alireza Heravi-Moussavi, Michelle Moksa, Matthew Mingay, Angela Hussainkhel, Brad Davis, Raman P. Nagarajan, Chibo Hong, Lorigail Echipare, Henriette O’Geen, Matthew J. Hangauer, Jeffrey B. Cheng, Dana Neel, Donglei Hu, Michael T. McManus, Richard Moore, Andrew Mungall, Yussanne Ma, Patrick Plettner, Elad Ziv, Ting Wang, Peggy J. Farnham, Steven J.M. Jones, Marco A. Marra, Thea D. Tlsty, Joseph F. Costello, Martin Hirst

**Affiliations:** 1 Department of Pathology, Helen Diller Family Comprehensive Cancer Center, University of California San Francisco, San Francisco, California 94143, USA; 2 Canada’s Michael Smith Genome Sciences Centre, BC Cancer Agency, Vancouver, British Columbia, Canada V5Z 1L3; 3 Centre for High-Throughput Biology and Department of Microbiology & Immunology, University of British Columbia, Vancouver, British Columbia, Canada V6T 1Z4; 4 Department of Neurological Surgery, Helen Diller Family Comprehensive Cancer Center, University of California San Francisco, San Francisco, California 94143, USA; 5 The Genome Center, University of California-Davis, Davis, California 95616, USA; 6 Department of Microbiology and Immunology, UCSF Diabetes Center, Department of Medicine, University of California San Francisco, San Francisco, California 94143, USA; 7 Department of Dermatology, University of California San Francisco, San Francisco, California 94143, USA; 8 Department of Medicine, Institute for Human Genetics and Helen Diller Family Comprehensive Cancer Center, University of California San Francisco, San Francisco, California 94115, USA; 9 School of Medicine, Department of Genetics, Washington University in St Louis, St Louis, Missouri 63108, USA; 10 Department of Biochemistry & Molecular Biology, Norris Comprehensive Cancer Center, University of Southern California, Los Angeles, California 90033, USA; 11 Department of Medical Genetics, University of British Columbia, Vancouver, British Columbia, Canada V6H 3N1

## Abstract

While significant effort has been dedicated to the characterization of epigenetic changes associated with prenatal differentiation, relatively little is known about the epigenetic changes that accompany post-natal differentiation where fully functional differentiated cell types with limited lifespans arise. Here we sought to address this gap by generating epigenomic and transcriptional profiles from primary human breast cell types isolated from disease-free human subjects. From these data we define a comprehensive human breast transcriptional network, including a set of myoepithelial- and luminal epithelial-specific intronic retention events. Intersection of epigenetic states with RNA expression from distinct breast epithelium lineages demonstrates that mCpG provides a stable record of exonic and intronic usage, whereas H3K36me3 is dynamic. We find a striking asymmetry in epigenomic reprogramming between luminal and myoepithelial cell types, with the genomes of luminal cells harbouring more than twice the number of hypomethylated enhancer elements compared with myoepithelial cells.

## Abstract (web summary)

Epigenetic changes associated with post-natal differentiation have been characterized. Here the authors generate epigenomic and transcriptional profiles from primary human breast cells, providing insights into the transcriptional and epigenetic events that define post-natal cell differentiation *in vivo*.

## Graphical abstract

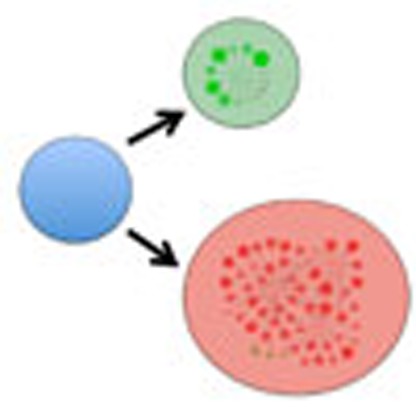

Our current concept of epigenetic patterning suggests a model where sequence-specific DNA-binding proteins act in concert with epigenetic regulatory cofactors to orchestrate a process of cellular differentiation accompanied by ordered and directional lineage restriction. Studies that have examined epigenetic lineage restriction have largely focused on the earliest stages of cellular differentiation where pluripotent potential is lost^1^,^2^. The extent to which these prenatal epigenetic processes are maintained during the end-stages of post-natal differentiation is largely unknown. Since epithelial cells within breast tissue, under the influence of hormones, mature post-natally to acquire their functionally different phenotypes, they present optimal cell sets to study the post-natal epigenetic changes.

The human breast is composed of ductal epithelial structures surrounded by various stromal components, consisting primarily of adipocytes, fibroblasts, immune cells, blood vessels and extracellular matrix^3^. Mammary ducts consist of myoepithelial, luminal epithelial (luminal) and rare progenitor cell types. Cell type-specific surface markers allow for the purification of these mammary cell populations that are enriched with functional and phenotypic characteristics of the *in vivo* cell type^4^. Breast myoepithelial and luminal cells are generated by bipotent or unipotent differentiation of resident mammary progenitor cells^5^ and thus provide a framework to investigate epigenetic changes that accompany normal post-natal epithelial differentiation.

We utilized this framework to examine the molecular events that accompany post-natal differentiation by generating epigenomic and transcriptional profiles from primary human breast cell types isolated from disease-free human subjects. From these data we define a comprehensive human breast transcriptional network including microRNAs (miRNAs), long noncoding RNAs and coding gene isoforms. We find that the degree of intron retention increases as cells near an end-stage differentiation state and we define a set of myoepithelial- and luminal epithelial-specific intronic retention events. Intersection of epigenetic states with RNA expression from distinct breast epithelium lineages demonstrates that mCpG provides a stable record of exonic and intronic usage, whereas H3K36me3 is dynamic. Our analysis also reveals a striking asymmetry in epigenomic reprogramming between luminal and myoepithelial cell types, which arise from a common progenitor. The genomes of luminal cells harbour more than twice the number of hypomethylated enhancer elements compared with myoepithelial cells and, although the transcriptional complexity of the two cell types is similar, the overall transcriptional output of luminal cells is ~4 times that of myoepithelial cells. Overall, our analysis provides a comprehensive view of highly purified genetically matched normal human mammary cell types and insights into the novel transcriptional and epigenetic events that define breast cell differentiation *in vivo*.

## Results

### Epigenomic profiling

**Figure 1:**
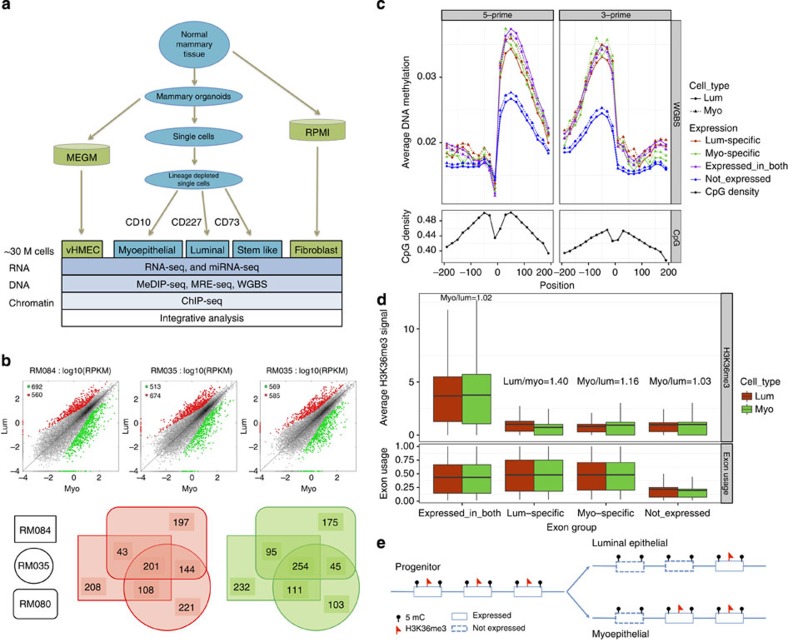
Differential expression and isoform analysis. (**a**) Experimental overview (**b**) DE genes in myoepithelial and luminal (lum) epithelial cell types across three donors, luminal upregulated (red), myoepithelial (myo) upregulated (green). (**c**) Exon–intron junction mCpGs provide an inherited signature of exon expression. Average number of CpGs (black, bottom panel) and average mCpG levels (whole-genome bisulphite shotgun, 20 bp bins) at exon junctions +/− 200 bp in luminal (solid line with round dots) and myoepithelial (dashed line with triangles). Exons are divided into four groups namely: (1) exons expressed in both the cell types (exon reads per kilobase of transcript per million reads mapped (RPKM) >0.1 in luminal and myoepithelial RM084, purple); (2) luminal-specific exons (isoform exons expressed in luminal but not in myoepithelial, red); (3) myoepithelial-specific exons (isoform exons expressed in myoepithelial but not in luminal, green) and (4) exons not expressed in either cell types (all other exons, blue). A statistical significant difference was observed between the not expressed exons and all other groups, *t*-test *P* value <10^−18^. All other comparisons show weak or no statistically significant difference. (**d**) H3K36me3 density in exon bodies provides a transient record of exon expression. Average H3K36me3 signal levels for exons in expressed genes (gene RPKM >0.1) in luminal RM080 (red) and myoepithelial RM080 (green). Exons are broken down into four groups namely: (1) exons expressed in both the cell types (exon RPKM >0.1 in luminal and myoepithelial); (2) luminal-specific exons (isoform exons expressed in luminal but not in myoepithelial); (3) myoepithelial-specific exons (isoform exons expressed in myoepithelial but not in luminal); and (4) exons not expressed in either cell types (all other exons). Fold enrichment of average H3K36me3 signal levels within exons revealed an increase H3K36me3 signals in cell type-specific exons in corresponding cell populations. (**e**) Model of inherited and transient epigenetic exon marking. Exon boundary DNA methylation (black dot) and exon body H3K36me3 (red flag) marking of exons in luminal and myoepithelial cell populations.

To explore the chromatin landscape of mammary cells, morphologically normal breast tissue obtained from disease-free women was dissociated and depleted of lineage-positive (primarily stromal) cells including endothelial cells, immune cells and fibroblasts (Fig. 1a and Methods). The remaining (primarily epithelial) cell population was subjected to fluorescence-activated cell sorting to 95% purity with established markers for myoepithelial and luminal epithelial (luminal); CD10 and MUC1, respectively^6^,^7^. In addition, we purified a population of CD73-expressing cells previously demonstrated to contain a rare cell population (~1–3%) with stem-like properties^8^ (stem like; Fig. 1a and Methods). Variant human mammary epithelial cells (vHMEC) and mammary fibroblasts were obtained *de novo* from cultured mammary tissue as previously described^9^,^10^. Massively parallel sequencing-based assays were employed to comprehensively annotate the expression and epigenetic states of these cell types (Fig. 1a and Supplementary Data 1 and Methods).

### Breast cell type-specific gene and exon expression

Unsupervised clustering of mRNA-seq derived gene and exon-level expression values revealed expected clustering by cell type (Supplementary Fig. 1). We observed an average of 1,211 differentially expressed (DE) genes (Fig. 1b and Supplementary Data 2) and 2,349 DE gene isoforms (Supplementary Fig. 2 and Supplementary Data 3; 90.5% validation rate, Supplementary Fig. 3) in equal proportions (up versus down) between the myoepithelial and luminal cell populations isolated from three individuals. A significant fraction of DE genes (455; hypergeometric *P* value=0) and isoforms (2,200; hypergeometric *P* value=0) overlapped across the three individuals and included previously documented^11^ (Supplementary Fig. 4) and novel myoepithelial- and luminal-specific transcriptional markers (Supplementary Data 2) that were confirmed by immunohistochemistry (IHC) on matched formalin-fixed, paraffin-embedded breast tissue sections (Supplementary Fig. 5).

### Epigenetic signature of exon usage

Epigenetic modifications, including mCpG and H3K36me3, are enriched within actively transcribed gene bodies and have been previously correlated with exon usage^12^,^13^. We confirmed a general correlation between DNA methylation and exon usage (Fig. 1c; two-sided *t*-test *P* value <10^−18^). However, surprisingly, when we restricted our analysis to cell type-specific exons (8,630 exons), mCpG levels between the myoepithelial and luminal cell populations showed no significant differences (Fig. 1c; two-sided *t*-test *P* value >0.05). Comparison of mCpG levels and exon usage between breast cell types and human embryonic stem cells (hESC) further supported this observation, where exons exclusively expressed in hESCs were found to retain a mCpG signature in myoepithelial and luminal epithelial cells (Supplementary Fig. 6; two-sided *t*-test *P* value <10^−4^). H3K36me3 levels were found to correlate with exon usage and, in contrast to mCpG levels, we observed an increase in H3K36me3 levels for cell type-specific exons (Fig. 1d; two-sided *t*-test *P* value=10^−25^ and Supplementary Fig. 7). Taken together, these results confirm that utilized exons generally have increased levels of mCpG and H3K36me3 and suggest that once mCpG is established at an exon–intron boundary, it is stably inherited through subsequent differentiations regardless of its expression. In contrast, H3K36me3 is dynamically gained and lost as a consequence of expression in a given cell type (Fig. 1e).

### Intronic retention levels increase in post-natal cell types

**Figure 2 f2:**
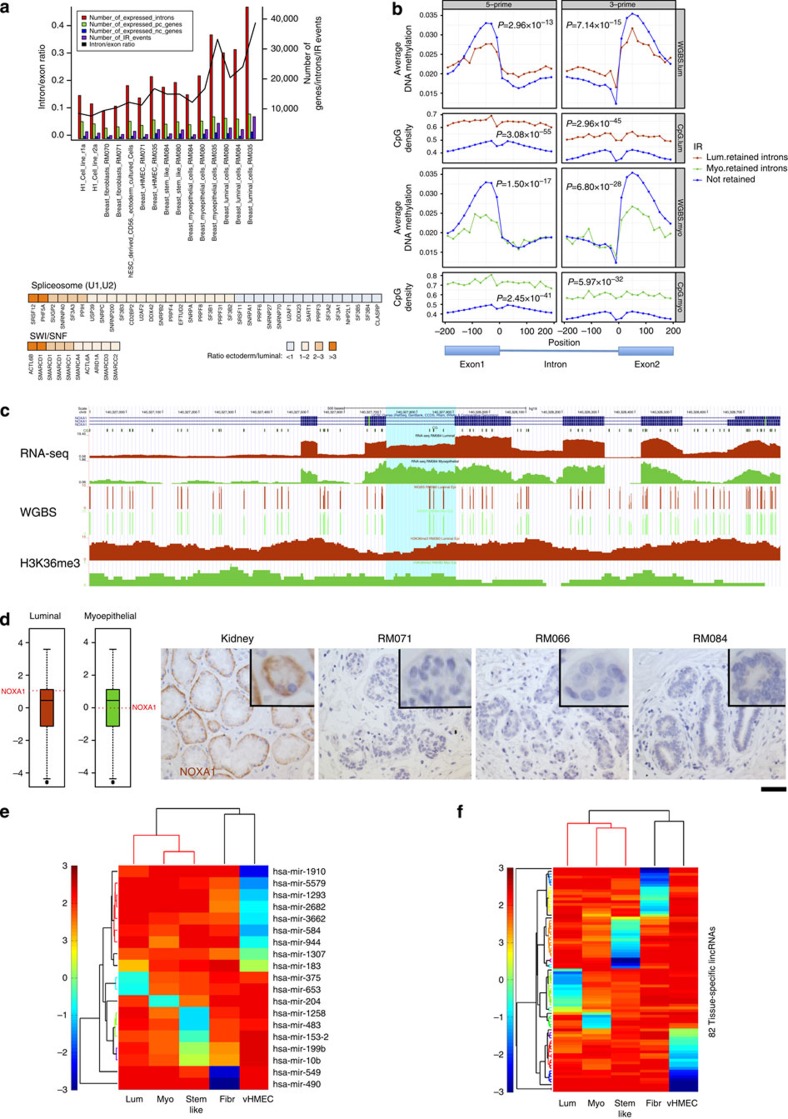
Intron retention and mammary cell type-specific miRNAs and long noncoding RNAs. (**a**) Upper panel—enumeration of expressed protein coding (pc) and noncoding (nc) genes, expressed introns and intron/exon ratio from hESCs (left) to luminal (lum) epithelial cells (right). Expression is defined by an RPKM >1. Lower panel—mRNA expression of U1 and U2 spliceosome subunits and SWI/SNF complex components in hESC derived CD56+ ectoderm cultured cells compared with RM084 luminal cells. (**b**) DNA methylation profile at intron boundaries. Average DNA methylation level profile at intron 5′ and 3′ +/− 200 bp with 20-bp bins in luminal RM066 (top panel) and myoepithelial RM045 (third panel) WGBS libraries. Average number of CpGs (second and fourth panel). Introns are divided into retained introns (red in luminal and green in myoepithelial (myo)) and not retained introns (blue) according to intron retention analysis in RM084. Differences in DNA methylation level across exon–intron boundaries are calculated by subtracting minimum DNA methylation level within introns (intron 5′+200 bp or 3′−200 bp, valley) from maximum DNA methylation level within exons (intron 5′−200 bp or 3′+200 bp, peak) and *P* values of *t*-test between retained introns and not retained introns are shown in DNA methylation panels. *P* values for CpG density panels are calculated by *t*-test on average No. of CpGs across the 400-bp boundaries between retained introns and not retained introns. (**c**) Visualization at UCSC genome browser of NOXA1 retained intron event (hg19 chr9:140327716–140327904—highlighted in blue) in luminal epithelial (red tracks) and myoepithelial cells (green tracks) across different assays: RNA-seq (RPKM values), WGBS-seq (CpG fractional methylation) and H3K36me3 ChIP-seq signal track. The `CG' track shows the location of CpG dinucleotides. (**d**) Boxplots show NOXA1 expression level (log10(RPKM)) in luminal epithelial (left) and myoepithelial (right) cell types. Validation of the loss of detectable NOXA1 protein in both breast cell types across RM071, RM066 and RM084 individuals with IHC compared to kidney used as a positive control. Scale bar: 50 μm. (**e**) Entropy heatmap of cell type-specific miRNAs across mammary cell types. (**f**) Entropy heatmap of cell type-specific lincRNAs (Supplementary Data 7) across mammary cell types. Fibr, fibroblast.

Intron retention is a widely observed but poorly studied phenomenon in both normal and transformed breast tissue^14^, where incomplete splicing leads to the retention of intronic sequences from pre-messenger RNAs that can target the resulting transcript for nonsense-mediated decay^15^. We found evidence for 3,693 retained introns in myoepithelial and luminal epithelial cell types of which the majority (67%) was cell type-specific using our thresholds (Supplementary Data 4). Strikingly, we found that the degree of intronic retention increased with differentiation state (Fig. 2a and Supplementary Fig. 8). Within breast cell types, luminal epithelial cells were found to express the largest number of intronic retained mRNAs with >7 times the number of events observed in myoepithelial cells (2,166 versus 304, respectively). To provide a possible mechanistic explanation, we examined the expression of the splicesome and SWI/SNF complex members and found that, similar to myeloid differentiation^15^, their expression was almost exclusively downregulated during differentiation (Fig. 2a lower panel). Retained introns were found to have increased CpG density overall (Fig. 2b; two-sided *t*-test *P* value <10^−30^), and we observed a reduction (two-sided *t*-test *P* value <10^−13^) in the degree of mCpG changes across exon–intron boundaries for retained introns. This observation supports a link between exon–intron boundary DNA methylation and exon splicing, which would not be expected to occur to the same degree when the flanking intron is retained (Fig. 2b and Supplementary Fig. 9). We also observed an increase in DNA methylation within retained introns. This effect was more pronounced in luminal epithelial cells, in which we also observed higher intron retention rates and expression levels, suggesting that this phenotype was not simply a reflection of an increase in CpG density. We validated a subset of intronic retention events by quantitative reverse transcription PCR (Supplementary Figs 10 and 11). Functional analysis of the intron-retained genes showed significant enrichment for genes encoding ‘phosphoproteins’ in both the cell types (Luminal; Benjamini *P* value <10^−31^ and Myoepithelial; Benjamini *P* value <10^−23^). As previously reported in granulocytes^15^, we found that the majority (90%) of intronic events resulted in mRNAs that would be predicted to undergo nonsense-mediated decay. In support of this prediction, we confirmed the absence of detectable protein by IHC for the *NOXA1* transcript (Fig. 2c). Taken together, these results suggest that intron retention may play a role in defining mammary cell types, particularly luminal cells, and, more generally, that intronic retention may be a common post-natal regulatory mechanism of gene expression.

### Breast cell type-specific noncoding RNA

Noncoding RNAs are key regulators of diverse cellular processes^16^ that can interact directly with the epigenetic machinery and may be prognostic in breast cancer^17^. We identified 936 unique miRNAs expressed at similar distributions across the five mammary-derived cell types, including a core set of 29 that were highly expressed (>1,000 reads per million (RPM)) across myoepithelial, luminal epithelial and stem-like cell types (Supplementary Fig. 12b and Supplementary Data 5). Hierarchical clustering demonstrated expected cell type relationships (Supplementary Fig. 12c), and cell type-specific miRNAs were identified with a majority being expressed in vHMECs (Fig. 2e). We also identified 1,870 expressed lincRNAs (Supplementary Fig. 13 and Supplementary Data 6) and 82 cell type-specific lincRNAs across the mammary cell types with myoepithelial cells showing the smallest number of cell type-specific events (Fig. 2f and Supplementary Data 7). Restricting our comparison to myoepithelial and luminal cells, we identified 206 DE noncoding RNAs, including 130 lincRNAs and 76 antisense transcripts. Among the DE lincRNAs, MALAT (NEAT2), a critical regulator of metastasis in epithelial cancers^18^, was overexpressed in normal luminal cells suggesting that its expression is not solely restricted to the metastatic potential in epithelial lineages. An imprinted region of 14q32.3, that encodes maternally expressed noncoding *MEG3* and *MEG8* transcripts and 54 miRNAs expressed from the maternally inherited homologue, was transcriptionally silenced in luminal cells (Supplementary Fig. 14). Loss of expression of the *MEG3* cluster through loss of heterozygosity and promoter hypermethylation is frequent in epithelial cancers^19^. Our results suggest that *MEG3* transcriptional repression is associated with normal epithelial differentiation and provide a novel intergenic differentially methylated region (DMR) that may be responsible for its cell type-specific regulation (Supplementary Fig. 14).

### DNA methylation defines a breast cell regulatory network

To discover putative regulatory elements including actively bound enhancers^20^, we identified 26,601 and 53,751 DNA unmethylated regions (UMR) in myoepithelial and luminal epithelial cells from whole-genome bisulphite data sets, respectively^21^ (Supplementary Fig. 15 and Supplementary Data 8). We validated these UMRs against orthologonal MeDIP-seq and MRE-seq data sets (Supplementary Fig. 16). Nearest gene-based analysis^22^ of the UMRs revealed highly significant enrichment for the expected pathways, including smooth muscle contraction (binomial false discovery rate (FDR) *Q* value=10^−71^) for myoepithelial cells and mammary gland epithelium development (binomial FDR *Q* value=10^−72^) for luminal epithelial cells (Supplementary Fig. 17). The UMRs overlapped almost exclusively with enhancer chromatin states (Supplementary Fig. 18), displayed significant overlap with ENCODE transcription factor (TF) chromatin immunoprecipitation (ChIP)-seq regions (luminal epithelial: 58%, myoepithelial: 60%, with ~20% expected by chance), and intersection with genome-wide association study alleles revealed a direct overlap with 10 single-nucleotide polymorphisms recently associated with the breast cancer risk loci (Supplementary Fig. 19, Supplementary Data 9), suggesting that these UMR regions are highly enriched in regulatory elements that define the breast cell types.[Fig f2]

### Regulatory asymmetry across breast cell types

**Figure 3:**
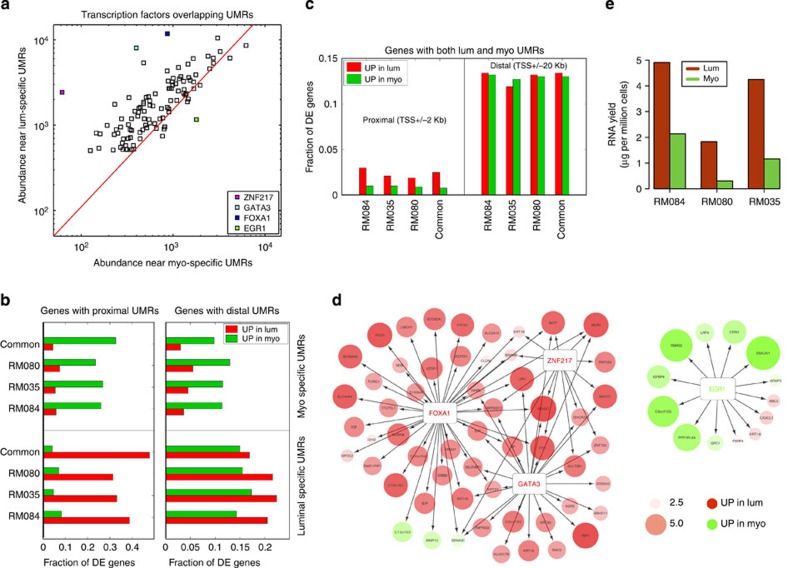
Regulatory asymmetry between myoepithelial and luminal cell types. (**a**) Plot of the relative abundance of TF binding sites overlapping UMRs in luminal (lum) and myoepithelial (myo) cells reveals regulatory asymmetry between the breast cell types. Red line represents total UMR abundance. (**b**) Fraction of DE genes (upregulated in luminal: red, upregulated in myoepithelial: green) associated with luminal (bottom panel) and myoepithelial (top panel) UMRs. Left panel shows proximal UMRs (UMRs within transcriptional start site (TSS) +/− 2 kb), and right panel shows distal UMRs (TSS +/− 20 kb). (**c**) Fraction of DE genes (upregulated in luminal: red, upregulated in myoepithelial: green) associated with both luminal and myoepithelial UMRs. Left panel shows proximal UMRs (UMRs within TSS +/− 2 kb) and right panel shows distal UMRs (TSS +/− 20 kb). (**d**) Differential expression (upregulated in luminal: red, upregulated in myoepithelial: green) of proximal UMRs overlapping with binding sites of luminal enriched (FoxA1, Gata3 and Znf217), and myoepithelial enriched TFs (Egr1). The size and shade of the circles represents fold change between luminal and myoepithelial gene expression on the log2 scale. (**e**) RNA yield (microgram per million cells) from luminal (red) and myoepithelial (green) cells extracted from three individuals.

Strikingly, we observed an asymmetry in the number of regulatory UMRs between breast cell types with 51 transcription factors having at least two times more sites in luminal than myoepithelial UMRs, while no TFs were more abundant in myoepithelial UMRs (Supplementary Data 10). The top three TFs (FOXA1, GATA3 and ZNF217) ranked by abundance of UMR-defined regulatory elements, are critical regulators of luminal cell biology and were at least eight times more abundant in luminal versus myoepithelial cells (Fig. 3a). To explore how these regulatory elements could influence the breast cell transcriptional programme, we associated them with genes and found a highly significant overlap (81%) between the cell type-specific UMRs and proximal upregulated DE genes (Fig. 3b). Interestingly, this association was highly directional with the majority (90.3%) of proximal UMRs associated with increased transcription in their respective cell types (Fig. 3b,d). This directionality was reduced for distal UMRs. Only 6% of the commonly expressed genes were found to be associated with cell type-specific proximal UMRs, highlighting their importance in defining a cell type-specific transcriptional programme (Fig. 3c).

The regulatory asymmetry observed between luminal and myoepithelial cells prompted us to examine the overall transcriptional output of these two cell types. While luminal cells demonstrated a higher overall intronic retention rate (Fig. 2a), we did not observe significant differences in the degree of gene association or in the number and type of expressed transcripts (including TFs) in the two breast cell types. Notably, however, normalization inherent to RNA-seq analysis masks the ability to determine the differences in the total transcriptional output between the cell types^23^. Therefore, to examine this effect directly, we calculated per cell RNA yields across myoepithelial and luminal cell populations extracted from three human subjects and found that luminal cells expressed 3.7+/− 1.3 times the amount of total RNA compared with myoepithelial cells (Fig. 3e). Taken together, these results reveal a striking regulatory asymmetry between myoepithelial and luminal cells that is correlated with transcriptional amplification in luminal cells. While transcriptional amplification has been demonstrated in c-MYC driven tumour cell types^24^, to our knowledge this is the first reported case of such an event occurring as a product of normal cellular differentiation.

## Discussion

Our analysis of primary normal human breast cell types has revealed novel insights into the transcriptional and epigenetic events that specify them. We provide a comprehensive database of the DE transcripts including isoforms for purified populations of normal breast epithelium, fibroblasts and derived vHMECs. Intersection of exon expression with epigenetic states confirmed that utilized exons generally have increased levels of mCpG and H3K36me3 and suggested that once mCpG is established at an exon–intron boundary it is stably inherited through subsequent differentiations, whereas H3K36me3 is transient. This pattern is consistent with a model where mCpG and H3K36me3 accumulate at the exon boundaries as a consequence of RNA polymerase pausing, and thus local enrichment of SETD2 (ref. 25) and DNMT3b (ref. 26), at the intron/exon boundaries^27^. Once established mCpG would be expected to be relatively stable through cell divisions, whereas H3K36me3 would be actively or passively lost.

Our analysis also revealed a potential role for intronic retention during breast cell differentiation, particularly in luminal cells, and, more generally, that intronic retention may be a common post-natal regulatory mechanism. We provide a comprehensive database of noncoding transcripts and observe that the imprinted *MEG3* locus is bi-alleically silenced during normal epithelial differentiation and provide evidence for a novel intergenic DMR that may responsible for its cell type-specific regulation.

We define a regulatory network for luminal and myoepithelial cells and provide an intersection with breast cancer genome-wide association study alleles. We found that a majority of regulatory elements could be associated with cell type-specific transcripts and that this association was highly directional, with >90% of proximal UMRs associated with increased transcription in their respective cell types. In this context, we observed a striking regulatory asymmetry between myoepithelial and luminal cells and correlated this with an overall increase in the cell-normalized transcriptional output in luminal cells^24^.

Taken together, our findings provide novel mechanistic clues about the events that contribute to normal epithelial differentiation and provide a comprehensive reference for future studies of disease.

## Methods

### Human tissue samples

Human breast tissues, obtained from disease-free women undergoing reduction mammoplasty after informed consent, were provided by the Cooperative Human Tissue Network (Nashville, TN) and the Kaiser Foundation Research Institute (Oakland, CA). Samples were identified through unlinked codes in accordance with the federal Health Insurance Portability and Accountability Act (HIPAA) guidelines. The information obtained about human tissue samples, including age, ethnicity and parity/gravidity, met the HIPAA guidelines. All tissues used in this study were devoid of visible disease, bacterial, fungal or viral contamination and exhibited a normal diploid 46, XX karyotype. Accrual and use of the breast tissues to conduct the studies described above were approved by the UCSF Committee on Human Research under Institutional Review Board protocol No. 10–01563.

### Dissociation of breast tissue

Breast tissue was dissociated mechanically and enzymatically, as previously described^8^,^9^. In brief, tissue was minced and dissociated in Roswell Park Memorial Institute medium (RPMI) 1640 with L-glutamine and 25 mM Hepes (Fisher; cat no. MT10041CV) supplemented with 10% (v/v) fetal bovine serum (FBS; JR Scientific; cat no. 43603), 100 units ml^−1^ penicillin, 100 μg ml^−1^ streptomycin SO4, 0.25 μg ml^−1^ fungizone, gentamycin (Lonza; cat no. CC4081G), 0.88 mg ml^−1^ collagenase-2 (Worthington; cat no. CLS-2) and 0.40 mg ml^−1^ hyaluronidase (Sigma; cat no. H3506-SG) at 37 °C for 16 h. The cell suspension was centrifuged at 394 × *g * for 10 min followed by a wash with RPMI 1640/10% FBS. Clusters enriched in epithelial cells (referred to as organoids) were recovered after serial filtration through a 150-μm nylon mesh (Fisher; cat no. NC9445658) and a 40-μm nylon mesh (Fisher; cat no. NC9860187). The final filtrate contained the mammary stromal cells, consisting primarily of fibroblasts and endothelial cells. Following centrifugation at 290 × *g* for 5 min, the epithelial organoids and filtrate were frozen for long-term storage. To generate single-cell suspensions, epithelial organoids were further digested for 5 min in 0.5 g l^−1^ trypsin-0.2 g l^−1^ EDTA-0.58 g l^−1^ NaHCO3 and 1 min in dispase-DNAse I (Stem Cell Technologies; cat no. 7913 and 7900, respectively) then filtered through a 40-μm cell strainer (Fisher; cat no. 087711). Fibroblasts were generated by culture of the filtrate in RPMI 1640 supplemented with 10% FBS and antibiotics and fungicides^28^. vHMECs were generated by culturing epithelial organoids in mammary epithelial cell growth medium (MEGM - Lonza; cat no. CC-03051) supplemented with antibiotics and fungicides^9^.

### Karyotyping

Karyotyping was carried out at Molecular Diagnostic Services Inc. (San Diego, CA). In brief, primary breast cells obtained from the filtrate fraction described above were allowed to grow to 80% confluency. Mitotic division was arrested by treating cells with 75 ng ml^−1^ colcemid for 18.5 h. Following treatment, cells were collected with trypsin–EDTA, treated with a hypotonic solution and fixed in methanol/acetic acid. Metaphase spreads were prepared from the fixed cells and stained to observe chromosomal G bands. For each tissue sample, 20 metaphase spreads were counted, 5 of which were analysed and karyotyped (Fig. 1a). In total, six independent donor samples were karyotyped. All samples analysed yielded a diploid 46, XX karyotype.

### Cell cycle analysis

Cell cycle analysis was carried out as previously described^29^. In brief, an aliquot of primary cells similar to those used for karyotyping was metabolically labelled with 10 μM bromodeoxyuridine (BrdU) at 37 °C for 4 h, trypsinized and fixed with ice cold 95% ethanol. Fixed cells were incubated with 0.08% pepsin (w/v in 0.1 N HCl) at 37 °C for 25 min with frequent shaking. Nuclei were isolated after cell digestion with 0.08% pepsin (w/v in 0.1 N HCl) at 37 °C for 25 min. Isolated nuclei were stained with an anti-BrdU antibody coupled to fluorescein isothiocyanate diluted 1:5 in 150 mM NaCl, 10 mM Hepes, pH 7.4, 4% fetal calf serum, 0.1% sodium azide (IFA) buffer over ice and in the dark for 30 min. Nuclei were washed once with 1 ml IFA buffer+0.5% Tween-20 and incubated with IFA buffer+5 μg ml^−1^ RNAse+50 μg ml^−1^ propidium iodide at 37 °C for 15 min. Samples were placed on ice for 15 min and analysed by flow cytometry on a FACSAria Sorter (Becton Dickinson). All analysed events were gated to remove debris and aggregates.

### Flow cytometry staining for cell sorting

The single-cell suspension obtained as described above was stained for cell sorting with a cocktail of biotinylated antibodies for lineage markers, anti-CD2, -CD3, -CD16, -CD64 (BD Biosciences; cat no. 555325, 555338, 555405 and 555526), −CD31 (Invitrogen; cat no. MHCD3115), −CD45 and −CD140b (BioLegend; cat no. 304003 and 323604) to specifically remove hematopoietic, endothelial and leukocyte lineage cells. Cells were stained for 20 min at room temperature in PBS with 1% BSA, followed by washing in PBS with 1% BSA. Biotinylated primary antibodies were revealed with an anti-human secondary antibody labelled with streptavidin-Pacific Blue conjugate (Invitrogen; cat no. S11222). The lineage-positive and negative cell populations were sorted using a FACSAria II cell sorter (BD Biosciences). The lineage-positive population was discarded. The lineage-negative cell population was incubated simultaneously with three human-specific primary antibodies (anti CD10 labelled with Phycoerythrin (PE) coupled to Cy7 (BD Biosciences; cat no. 341092), anti-MUC1 labelled with fluorescein isothiocyanate (BD Biosciences; cat no. 559774) and anti-CD73 labelled with PE (BD Biosciences; cat no. 550257) for 20 min at room temperature in PBS with 1% BSA, followed by washing in PBS with 1% BSA. Cells were then sorted as described above.

### Immunohistochemistry

Human disease-free breast tissues were fixed in formalin and paraffin-embedded using a standard protocol. Paraffin-embedded tissues were cut into 4-μm serial sections, laid on Polysine microscope slides (Thermo Scientific, cat no. 6776215), deparaffinized and rehydrated using standard procedures. All steps were carried out at room temperature except when noted. Antigen retrieval was carried out by microwaving the tissue sections in citrate buffer, pH 6.0, for 10 min (for the detection of CD10, MUC1, claudin 4 and NOXA1) or in EDTA at pH 8.0 for 10 min (for the detection of anoctamin 1/TMEM16A). Sections were then incubated with either a primary mouse monoclonal antibody against CD10 (Thermo Scientific; cat no. MS-728-S0, clone 56C6) diluted 1/20 or a primary mouse monoclonal antibody against MUC1 (Upstate Biotechnology; cat no. 05–653, clone 232A) diluted 1/200 or a primary mouse monoclonal antibody against claudin 4 (Life Technologies; cat no. 18–7341, clone 3E2C1) diluted 1/200 or a primary rabbit polyclonal antibody against anoctamin 1/TMEM16A (Abcam; cat no. ab53212) diluted 1/1,600 or a primary rabbit polyclonal antibody against NOXA1 (Novus Biologicals; cat no. NBP1–92197) diluted 1/100 overnight at 4 °C. The staining was visualized after incubation with the HRP polymer kit (Ultravision LP kit, Thermo Scientific, cat no. TL-125 HLS) for 30 min according to the manufacturer’s instructions and with diaminobenzidine substrate (Genemed; cat no. 520017) for 5 min. Tissue sections were counterstained with hematoxylin and mounted. Stained sections were imaged at × 10, × 20 and × 40 magnification using a DS-Ri1 Nikon colour digital camera mounted to a DMLB Leica microscope.

### RNA sequencing

Standard operating procedures for RNA-seq library construction are available (http://www.roadmapepigenomics.org/protocols/type/experimental/) or by request. RNA-seq library construction involves the following standard operating procedures (SOPs) in order: (1) Purification of polyA+ mRNA and mRNA(−) flowthrough total RNA using MultiMACS 96 separation unit; (2) strand-specific 96-well complementary DNA (cDNA) synthesis; (3) strand-specific 96-well library construction for Illumina sequencing. In brief, polyA+ RNA was purified using the MACS mRNA isolation kit (Miltenyi Biotec, Bergisch Gladbach, Germany), from 2–10 μg of total RNA with a RIN >=7 (Agilent Bioanalyzer) as per the manufacturer's instructions. The process included on-column DNaseI treatment (Invitrogen, Carlsbad, CA, USA). Double-stranded cDNA was synthesized from the purified polyA+ RNA using the Superscript II Double-Stranded cDNA Synthesis kit (Invitrogen) and 200 ng random hexamers (Invitrogen). After first strand synthesis, dNTPs were removed using 2 volumes of AMPure XP beads (Beckman Genomics, Danvers, MA, USA). GeneAmp (Invitrogen) 12.5 mM dNTPs blend (2.5 mM dCTP, 2.5 mM dGTP, 2.5 mM dATP, 5.0 mM dUTP) was used in the second-strand synthesis mixture in the presence of 2 μg of ActinomycinD. Double-stranded cDNA was purified using 2 volumes of Ampure XP beads, fragmented using Covaris E series shearing (20% duty cycle, Intensity 5, 55 s), and used for paired-end sequencing library preparation (Illumina). Before library amplification uridine digestion was performed at 37 °C for 30 min following with 10 min at 95 °C in Qiagen Elution buffer (10 mM Tris–Cl, pH 8.5) with 5 units of Uracil-N-Glycosylase (UNG: AmpErase). The resulting single-stranded sequencing library was amplified by PCR (10–13 cycles) to add Illumina P5 and P7 sequences for cluster generation. PCR products were purified on Qiaquick MinElute columns (Qiagen, Mississauga, ON) and assessed and quantified using an Agilent DNA 1000 series II assay and Qubit fluorometer (Invitrogen, Carlsbad, CA), respectively. Libraries were sequenced using paired-end 76 nt sequencing chemistry on a cBot and Illumina GA_iix_ or HiSeq 2000 following the manufacture’s protocols (Illumina).

RNA-seq pair-end reads are aligned to a transcriptome reference consisting of the reference genome extended by the annotated exon–exon junctions^30^. To generate transcriptome reference we used the JAGuaR v 1.7.6 pipeline^31^. Reads aligned to a custom transcriptome reference (build from NCBI GRCh37-lite reference and Ensembl v65 (GenCode v10) annotations) were then ‘repositioned’ on to genomic coordinates, transforming reads that span exon–exon junctions into large-gapped alignments. Using repositioned reads we generated genome-wide coverage profiles (wiggled files) using a custom BAM2WIG java program (http://www.epigenomes.ca/tools.html) for further analysis and visualization in genome browsers. To generate profiles we included pairs that are marked as duplicated as well as pairs that are mapped in multiple genomic locations.

A custom RNA-seq QC and analysis pipeline was applied to the generated profiles and a number QC metrics were calculated to assess the quality of RNA-seq library such as intron–exon ratio, intergenic reads fraction, strand specificity (for stranded RNA-seq protocols), 3′-5′ bias, GC bias and reads per kilobase of transcript per million reads mapped (RPKM) discovery rate (see Supplementary Data 11). To quantify the exon and gene expression we calculated modified RPKM metrics^32^. For the normalization factor in RPKM calculations we used the total number of reads aligned into coding exons and excluded reads from mitochondrial genome as well as reads falling into genes coding for ribosomal proteins as well as reads falling into top 0.5% expressed exons. RPKM for a gene was calculated using the total number of reads aligned into its all merged exons normalized by total exonic length. The resulting files contain RPKM values for all annotated exons and coding and noncoding genes, as well as introns (Supplementary Data 12 and 13). We also report the coordinates of all significant intergenic RNA-seq clusters not overlapping the annotated genes.

Exon-level-normalized RPKM were used to identify cell type-specific isoforms (Supplementary Data 14). Pairwise comparisons between different cell types within the same individual were performed to identify DE exons using a custom DEfine matlab tool (FDR cutoff=0.015). Expressed exons were defined as those with RPKM ≥10% of gene RPKM; unexpressed exons were defined as those with RPKM ≤1% of gene RPKM; and the exons in between (1~10% gene RPKM) were discarded to filter out most false positives. Isoforms for each pairwise comparison were identified as genes with DE exons expressed in only one of the two samples. DE genes were excluded from this list. Functional analysis of myoepthelial and luminal isoforms revealed the enrichment of genes encoding proteins involved in intracellular signalling (Supplementary Fig. 20), while no functional enrichment was found for the individual-specific isoforms (Supplementary Data 3). In addition, exon–exon junction coverages were calculated from RNA-seq BAM files. On average, 38.6% of the identified isoforms demonstrated junction coverage due to the difficulty in placing sequence reads that span exon–exon junctions, and among them 87.3% isoform genes have support from junction reads (junction RPKM >0.1 in one sample and <0.1 in the other, Supplementary Data 3). For strand-specific libraries (RM084) in particular, we have much higher junction coverage (53.7%) and junction support (99.3%). Restricting our analysis to strand-specific libraries, we found that cassette exons are enriched gene boundaries ends (Supplementary Fig. 21), and that isoform genes are expressed at a lower level in general (Supplementary Fig. 22).

Intron-level-normalized RPKM and normalized coverage were used to identify retained introns in all cell/tissue types. Expressed introns were first prefiltered as those with RPKM >1. The retained introns were defined as those with RPKM >5% of the protein coding gene RPKM and with normalized coverage >30% of the flanking exons normalized coverage. Retained introns also have at least 90% of their sequence covered by at least one read. Introns that overlap exons on the opposite strand (18902 introns out of 257013) were excluded from the intron retention analysis to normalize across strand-specific and non strand-specific libraries.

### miRNA sequencing

Standard operating procedures for RNA-seq library construction are available (http://www.roadmapepigenomics.org/protocols/type/experimental/) or by request. miRNA-seq library construction involves the following SOPs in order: (1) Purification of polyA+ mRNA and mRNA(−) flowthrough total RNA using MultiMACS 96 separation unit; (2) miRNA3—plate format miRNA library construction. In brief, flowthrough total MultiMACS RNA was recovered by ethanol precipitation and arrayed into 96-well plates, with controls as described below. Flowthrough RNA quality was checked for a subset of samples using an Agilent Bioanalyzer RNA nano chip. An adenylated 3′ adapter was ligated using a truncated T4 RNA ligase2 (NEB Canada, cat. M0242L) with an incubation of 1 h at 22 °C. An RNA 5′ adapter was then added, using a T4 RNA ligase (Ambion USA, cat. AM2141) and ATP, and is incubated at 37 °C for 1 h. Following ligation 1st strand cDNA is synthesized using Superscript II reverse transcriptase (Invitrogen, cat.18064 014). The resulting product was PCR amplified using the 3′ PCR primer (5′-CAAGCAGAAGACGGCATACGAGAT-3′) and an indexed 5′ PCR primer (5′-AATGATACGGCGACCACCGACAGNNNNNNGTTCAGAGTTCTACAGTCCGA-3′), Phusion Hot Start High Fidelity DNA polymerase (NEB Canada, cat. F-540 l), buffer, dNTPs and dimethylsulphoxide (DMSO). PCR is run at 98 °C for 30 s, followed by 15 cycles of 98 °C for 15 s, 62 °C for 30 s and 72 °C for 15 s, and finally a 5 min incubation at 72 °C. Library quality was assessed on a Caliper LabChipGX DNA chip. PCR products were then pooled (up to 16 per pool) and size selected by agarose electrophoresis to remove larger cDNA fragments and smaller adapter contaminants. After size selection, each pool was ethanol precipitated, quality checked using an Agilent Bioanalyzer DNA 1000 chip and quantified using a Qubit fluorometer (Invitrogen, cat. Q32854). Each pool was then diluted to a target concentration for cluster generation and loaded into a single lane of an Illumina GA_IIx_ or HiSeq 2000 flow cell. Clusters are generated, and lanes sequenced with a 31-bp main read for the insert and a 7-bp read for the index. The resulting sequences were separated into individual samples based on the index read sequences and assessed for quality. Adapter sequence was then trimmed off using custom scripts, and the trimmed reads for each sample were aligned to the NCBI GRCh37-lite reference genome using Burrows–Wheeler alignment (BWA) as described in ref. 33 (see Supplementary Data 5 and 15).

### ChIP sequencing

Standard operating procedures for ChIP-seq library construction are available (http://www.roadmapepigenomics.org/protocols/type/experimental/) or by request. ChIP-seq library construction involves the following SOPs in order: (1) Crosslinking of frozen cell pellet; (2) DNA sonication using Sonic Dismembrator 550; (3) SLX-PET protocol for Illumina sample preparation. Antibodies used in this study were subjected to rigorous quality assessment to meet the reference epigenome mapping quality standards (http://www.roadmapepigenomics.org/protocols) including western blot of whole-cell extracts, 384 peptide dot blot (Active Motif MODified Histone Peptide Array) and ChIP-seq using control cell pellets (HL60). Antibody vendor, catalogue number and lot are provided along with ChIP-seq library construction details as part of the metadata associated with all ChIP-seq data sets and available through GEO and the NCBI epigenomics portals (for example, http://www.ncbi.nlm.nih.gov/geo/query/acc.cgi?acc=GSM613886). Final library distributions were calculated using an Agilent Bioanalyzer and quantified by fluorometric quantification (Qubit, Life Technologies). Libraries were sequenced using single-end 76 nt sequencing chemistry on an Illumina GA_iix_ or HiSeq 2000 following the manufacturer’s protocols (Illumina) as either single or multiplex using custom index adapters added during library construction.

Raw sequences were examined for quality, sample swap and reagent contamination using custom in house scripts. Sequence reads were aligned to NCBI GRCh37-lite reference using BWA 0.5.7 (ref. 34) and default parameters, and assessed for overall quality using Findpeaks^35^. Aligned reads were directionally extended by the average insert size of the DNA fragments for a given library estimated from Agilent Bioanalyzer (Agilent) profiles measured during library construction and varied between ~130 and 250 bp. Custom java program (BAM2WIG) was used to generate wig files for downstream analysis and visualization. Reads with BWA mapping quality scores <5 were discarded and reads that aligned to the same genomic coordinate were counted only once in the profile generation.

### Methylated DNA immunoprecipitation sequencing

DNA (2–5  μg) was sonicated to ~100–500 bp with a Bioruptor sonicator (Diagenode). Sonicated DNA was end-repaired, A-tailed and ligated to single-end adapters following the standard Illumina protocol. After agarose sized-selection to remove unligated adapters, adapter-ligated DNA was used for each immunoprecipitation using a mouse monoclonal anti-methylcytidine antibody (1 μg ml^−1^, Eurogentec, catalogue no. BI-MECY-0100). DNA was heat-denatured at 95 °C for 10 min, rapidly cooled on ice, and immunoprecipitated with 1 μl primary antibody per microgram of DNA overnight at 4 °C with rocking agitation in 500 μl IP buffer (10 mM sodium phosphate buffer, pH 7.0, 140 mM NaCl and 0.05% Triton X-100). To recover the immunoabsorbed DNA fragments, 1 μl of rabbit anti-mouse IgG secondary antibody (2.5 μg ml^−1^, Jackson Immunoresearch) and 100 μl Protein A/G beads (Pierce Biotechnology) were added and incubated for an additional 2 h at 4 °C with agitation. After immunoprecipitation, a total of six IP washes were performed with ice cold IP buffer. A non-specific mouse IgG IP (Jackson Immunoresearch) was performed in parallel to methyl DNA IP as a negative control. Washed beads were resuspended in Tris-EDTA buffer (TE) with 0.25% SDS and 0.25 μg ml^−1^ proteinase K for 2 h at 55 °C and then allowed to cool to room temperature. MeDIP and supernatant DNA were purified using Qiagen MinElute columns and eluted in 16 μl EB (Qiagen, USA). Fifteen cycles of PCR were performed on 5 μl of the immunoprecipitated DNA using the single-end Illumina PCR primers. The resulting reactions were purified over Qiagen MinElute columns, after which a final size selection (192–392 bp) was performed by electrophoresis in 2% agarose. Libraries were quality controlled by spectrophotometry and Agilent DNA Bioanalyzer analysis. An aliquot of each library was diluted in EB to 5 ng μl^−1^ and 1 μl used as template in four independent PCR reactions to confirm the enrichment of methylated and de-enrichment of unmethylated sequences, compared with 5 ng of the input (sonicated DNA). Two positive controls (SNRPN and MAGEA1 promoters) and two negative controls (a CpG-less sequence on Chr15 and GAPDH promoter) were amplified. Cycling was 95 °C for 30 s, 58 °C for 30 s, 72 °C for 30 s with 30 cycles. PCR products were visualized by 1.8% agarose gel electrophoresis.

### Methylation sensitive restriction enzyme sequencing

Three parallel digests were performed (HpaII, AciI and Hin6I; Fermentas), each with 1 μg of DNA. Five units of enzyme per microgram DNA were added and incubated at 37 °C in Fermentas ‘Tango’ buffer for 3 h. A second dose of enzyme was added (five units of enzyme per microgram DNA) and the DNA was incubated for an additional 3 h. Digested DNA was precipitated with sodium acetate and ethanol, and 500 ng of each digest were combined into one tube. Combined DNA was size selected by electrophoresis on a 1% agarose Tris–borate–EDTA gel. A 100–300-bp gel slice was excised using a sterile scalpel and gel-purified using Qiagen Qiaquick columns, eluting in 30 μl of Qiagen EB buffer. Library construction was performed using the Illumina Genomic DNA Sample Kit (Illumina Inc., USA) with single-end adapters, following the manufacturer’s instructions with the following changes. For the end repair reaction, T4 DNA polymerase and T4 polynucleotide kinase were excluded and the Klenow DNA polymerase was diluted 1:5 in water, and 1 μl was used per reaction. For single-end oligo adapter ligation, adapters were diluted 1:10 in water, and 1 μl was used per reaction. After the second size selection, DNA was eluted in 36 μl EB buffer using Qiagen Qiaquick columns, and 13 μl was used as a template for PCR, using Illumina reagents and cycling conditions with 18 cycles. After cleanup with Qiagen MinElute columns, each library was examined by spectrophotometry (Nanodrop, Thermo Scientific, USA) and Agilent DNA Bioanalyzer (Agilent, USA).

### Calculation of MeDIP-seq scores for single CpGs

Raw MeDIP-seq sequences were examined for quality, sample swap and reagent contamination using custom in house scripts. Sequence reads were aligned to NCBI GRCh37-lite reference using BWA 0.5.7 (ref. 34) and default parameters. To transform aligned MeDIP-seq sequences to single CpG fractional calls for each library we calculated the MeDIP coverage signal for CpGs genome wide and the average coverage in all genomic regions, which were >=500 bp away from a CpG. The latter was used as a background. Next, we convert signal and background values into the MeDIP methylation score: a continuous value between 0 and 1 with a distribution very similar to the one of whole-genome bisulphite sequencing (WGBS) fractional methylation. During this process we exclude locations with mapability <0.5 and correct for mapability for the locations with mapability between 0.5 and 1. After MeDIP score assignment we assessed its specificity and sensitivity against WGBS data sets and derived thresholds for hypomethylated (<0.35) and hypermethylated (>0.8) scores. The remaining CpGs were considered to have intermediate methylation. These thresholds were used to analyse individual-specific and tissue-specific methylation patterns and detect DMRs. For detected DMRs we calculate the MRE signal (for the subset of CpGs where data are available) for cross validation.

### Whole-genome bisulphite sequencing

Qubit quantified genomic DNA (1–5 μg) was utilized for library construction. Unmethylated Lambda DNA (Promega, Cat no. D1521) was added to genomic DNA for a 0.1% final concentration. DNA was sonicated to a fragment size of ~300 bp using a Bioruptor sonicator (Diagenode). End-repair, addition of 3′ A bases and adapter ligation was performed as per the Illumina PE genomic DNA sample prep kit protocol except that methylated cytosine PE adapters were used. Bisulfite conversion of purified adapter-ligated DNA was performed using the EZ DNA Methylation Gold kit (ZymoResearch Cat.D5005) according to the manufacturer's instructions. The DNA was then purified with the Qiagen Qiaquick kit, followed by PCR enrichment using Kapa HiFi Hot Start Uracil+Ready (Kapa Biosystems, Cat no. KK2801) for five cycles with PCR PE primers 1.0 and 2.0. PCR products were purified with the Qiagen Minelute kit and size selected with PAGE gel purification. DNA libraries were checked for quantity by Qubit (Life Technologies) and quality by Agilent DNA Bioanalyzer (Agilent). Libraries were sequenced using paired-end 100 nt sequencing chemistry on an Illumina HiSeq 2000 following the manufacturer's protocols (Illumina).

Raw WGBS sequences were examined for quality, sample swap and reagent contamination using custom in house scripts. Sequence reads were directionally aligned to the human genome (GRCh37-lite) as described in ref. 36. UMRs were detected using C++ tool as described in ref. 21 with a *P* value <0.0005. The median size of the identified UMRs was ~270 bp with five (median) differentially methylated CpG per region.

### Entropy-based thresholds

We used an entropy-based strategy to identify cell type-specific expression events. In brief, for each comparison we have *N* measurements (*N* cell types) for a given gene (for example, RPKM for lincRNAs, RPM for miRs and so on): *E*_*i*(gene), with *i*=1..*N*. We introduce a variable for every gene called entropy (*H*) where *H*(gene)=−sum(*F*_*i* *log2(*F*_*i*)), where index '*i*' runs over all cell types *i*=1..*N*, and *F*=*E*_*i*(gene)/sum(*E*_*i*(gene)). When *H* is small ~0—the gene is expressed in just one cell type; if *H* is large and~log2(*N*)—this gene is expressed uniformly in all cell types. We introduce another quantity 'tissue specificity', for a given gene for each cell types: *Q*(gene)_*i*=*H*−log2(*F*_*i*). We next threshold our data in two-dimensional space: [*H*(gene), max(*E*_*i*(gene))] to ensure that the gene is expressed in at least one cell type and has small entropy. Thresholds are not independent; typically if gene has large expression, we have to allow higher entropy. We cluster *Q* variable for all genes that pass the threshold to generate heat maps.

### cDNA synthesis for validations

The quality and yield of the isolated RNA (seven samples plus Universal Human Reference total RNA control (Agilent Technologies, USA)) was assessed using Agilent 2100 Bioanalyzer (Agilent Technologies, Santa Clara, CA, USA). PolyA+ RNA was purified using the 96-well MultiMACS mRNA isolation kit on the MultiMACS 96 separator (Miltenyi Biotec, Germany) with on-column DNaseI treatment as per the manufacturer's instructions. The eluted PolyA+ RNA was ethanol precipitated and resuspended in 8 μl of Nuclease free water with 1:20 SuperaseIN (Life Technologies, USA). First strand cDNA was synthesized from the purified polyA+ RNA using the Maxima H Minus First Strand cDNA Synthesis Kit (Thermo Scientific, USA). Synthesis of the second-strand cDNA was carried out in 50-μl reaction volume using second-strand reagents from SuperScript double-stranded cDNA synthesis kit (Life Technologies, USA). Double-stranded cDNA was quantified on Qubit Fluorometer using the Qubit dsDNA HS assay (Life Technologies, USA). The quality of cDNA was assesed on the Agilent Bioanalyzer using the high sensitivity DNA chip assay.

### Intronic retention validation

Primers for the respective targets were designed using Primer3 (primer3.sourceforge.net). The thermodynamic suitability of the primer pairs was verified using the IDT Oligo Analyzer (Integrated DNA Technologies, USA) and *in silico* PCR was carried out using the UCSC genome browser to ensure primer specificity. Primers were synthesized by Integrated DNA Technologies, USA and resuspended to 100 μM stock with EB buffer (Qiagen, USA).

To validate the primer pairs to be used in the downstream qPCR analysis a 50 μl reaction was set up for each primer pair using 0.5 μl of Phusion HS II polymerase (2 U μl^−1^; Thermo Scientific), 10 μl of 5x HF buffer, 1.5 μl of DMSO, 1 μl of 10 mM dNTPs, 0.25 ng of cDNA template, 2 μl of forward primer (10 mM; Integrated DNA Technologies, USA) and 2 μl of reverse primer (10 mM; Integrated DNA Technologies, USA). Each reaction was topped up to 50 μl with Ultrapure water. The reactions were denatured at 98 °C for 1 min before 35 cycles at 98 °C (15 s)/64 °C (15 s)/72 °C (15 s) with a final extension for 5 min at 72 °C. For visualization, 10 μl from each PCR reaction was diluted 1:2 with Ultrapure water, loaded on a 1% Agarose E-gel (Invitrogen, USA) and run for 10 min.

The qPCR assay was performed in MicroAmp Optical 384-well plates (Applied Biosystems, USA) in a 10 μl reaction volume in triplicate for each target using ViiA 7 Real-Time PCR System with 384-well heating block (Life Technologies, USA). Each reaction contained: 5 μl of 2X SYBR Green PCR master mix (Life Technologies, USA), 4 μl of primer mix (final primer concentration 250 nM each) and 1 μl of cDNA template (0.25 ng μl^−1^). The qPCR run method consisted of 98 °C for 10 min (‘hot start’) followed by 40 cycles of 98 °C for 15 s (denaturation) and 62 °C for 1 min (gene target amplification).

The experimental suitability of each primer pair was verified by including non-template control samples (to check primer-dimer formation in the experimental conditions) and dissociation curves for the amplicons generated in the experimental samples. A final dissociation step was always performed at the end of each PCR assay to verify the unique and specific amplification of the target sequence.

Fold changes were calculated with 2-ΔCt formula, where ΔCt is Ct(Intron–Exon primers)—Ct(Exon–Exon primers). Fold changes were averaged across triplicates and the s.d. was computed (outlier Ct values were excluded from the mean calculation). Fold change was plotted against the intron retention level that is the ratio between intron-level-normalized coverage and flanking exon normalized coverage (ratio was computed for both the flanking exons and the averaged ratio is reported). Fold change values that are <1 were replaced by −2ΔCt in this plot (Supplementary Fig. 11) to aid interpretation.

### Isoform junction PCR validation

Primers flanking isoform junctions were designed using Primer3 (primer3.sourceforge.net). The thermodynamic suitability of the primer pairs was verified using the IDT Oligo Analyzer (Integrated DNA Technologies, USA) and *in silico* PCR was carried out using the UCSC genome browser to ensure primer specificity. Primers were synthesized by Integrated DNA Technologies (USA) and resuspended to 100 μM stock concentration with EB buffer (Qiagen, USA).

To validate the presence of the isoform junctions, a 50 μl PCR reaction was set up using primers flanking the junction under investigation. The reaction consisted of 0.5 μl of Phusion HS II polymerase (1 U; Thermo Scientific), 10 μl of 5x HF buffer, 1.5 μl of DMSO, 1 μl of 10 mM dNTPs, 0.5 ng of cDNA template, 2 μl of forward primer (10 mM; Integrated DNA Technology, USA) and 2 μl of reverse primer (10 mM; Integrated DNA Technologies, USA). Each reaction was topped up to 50 μl with Ultrapure water (Invitrogen, USA). Conditions for PCR amplification were as follows: 98 °C for 1 min then 35 cycles at 98 °C (15 s)/63 °C (15 s)/72 °C (15 s) followed by a final extension for 5 min at 72 °C. For visualization, 10 μl from each PCR reaction was diluted 1:2 with Ultrapure water, loaded onto a 1% Agarose E-gel (Invitrogen, USA) and run for 10 min.

## Additional information

**How to cite this article:** Gascard, P. *et al*. Epigenetic and transcriptional determinants of the human breast. *Nat. Commun.* 6:6351 doi: 10.1038/ncomms7351 (2015).

## Supplementary Material

Supplementary Figures Supplementary Figures 1-22

Supplementary Data 1 Summary of molecular libraries and accessions

Supplementary Data 2 Cell-type specific genes. Genes that are UP (DOWN) regulated in luminal epithelilal vs myo epithelilal commonly in three comparisons for RM080, RM035 and RM084. Gene lists are based on an FDR = 0.015 threshold with a minimal RPKM > 0.005 and minimal read count per gene > 25.

Supplementary Data 3 List of cell type-specific isoforms identified by RNA-seq analysis. Tab 1 is the summary of No. of isoforms identified and validated with junctions. Tab 2 is the summary of No. of isoforms between luminal and myoepithelial across three individuals. Tabs 3-8 list gene isoforms identified between pairwise comparisons between different cell types.

Supplementary Data 4 Myoepithelial and luminal epithelial gene intronic retention events.

Supplementary Data 5 RPM (reads per million) for breast cells miRNAs.

Supplementary Data 6 lincRNAs expressed in breast cell populations. RPKM values are reported for lincRNA using Ensembl v73 annotations. Values for the fibroblast RM070 and RM071 were averaged.

Supplementary Data 7 List of lincRNAs for every cluster in Figure 2e.

Supplementary Data 8 Unmethylated regions (UMRs) in myoepithelial and luminal cell types called from whole genome bisulfite datasets.

Supplementary Data 9 List of GWAS SNPs associated breast cancer risk loci that overlap with luminal and myoepithelial UMRs.

Supplementary Data 10 Transcription factors for which ENCODE ChIP-seq binding locations overlap significantly with UMR regions.

Supplementary Data 11 Summary quality controls metrics for RNA-seq libraries included in this study.

Supplementary Data 12 Ensembl v65 protein coding RPKM expression matrix

Supplementary Data 13 Ensembl v65 non-coding RPKM expression matrix

Supplementary Data 14 Ensembl v65 exon-level RPKM expression matrix

Supplementary Data 15 Annotation priorities used to resolve multiple database matches for a single alignment location and multiple alignment locations for a read.
