# Supplementary material for: Epigenetic and transcriptional determinants of the human breast
Source: Nat Commun. 2015 Feb 18;6:6351. doi: 10.1038/ncomms7351 (PMC4346612; doi:10.1038/ncomms7351)
Supplement: Supplementary Figures — 1-22 [file ncomms7351-s1.pdf]

Supplementary Information

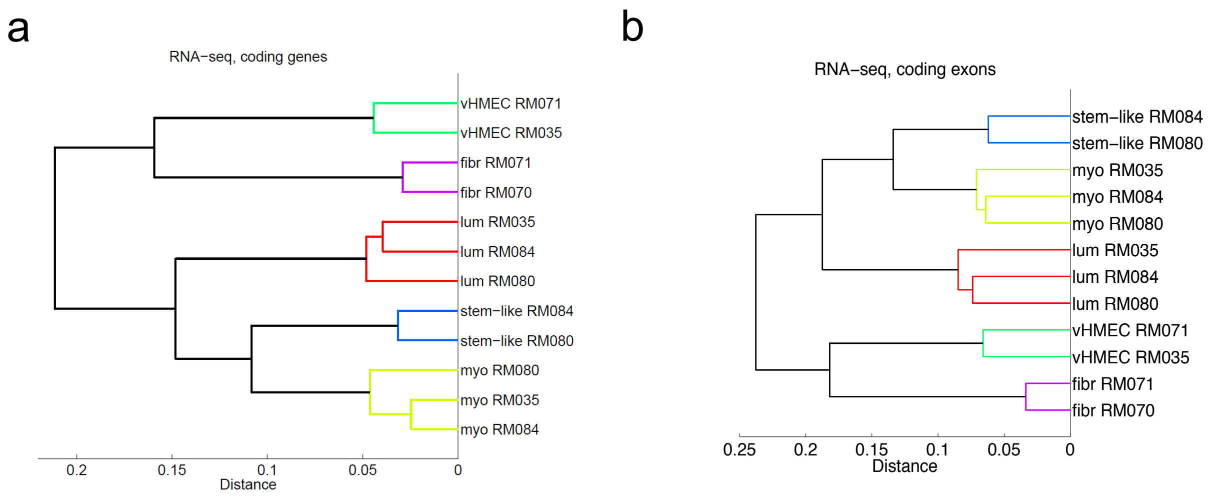

Supplementary Figure 1. Gene-level (**a**) and exon-level (**b**) unsupervised clustering reveals breast derived cell-type relationships.

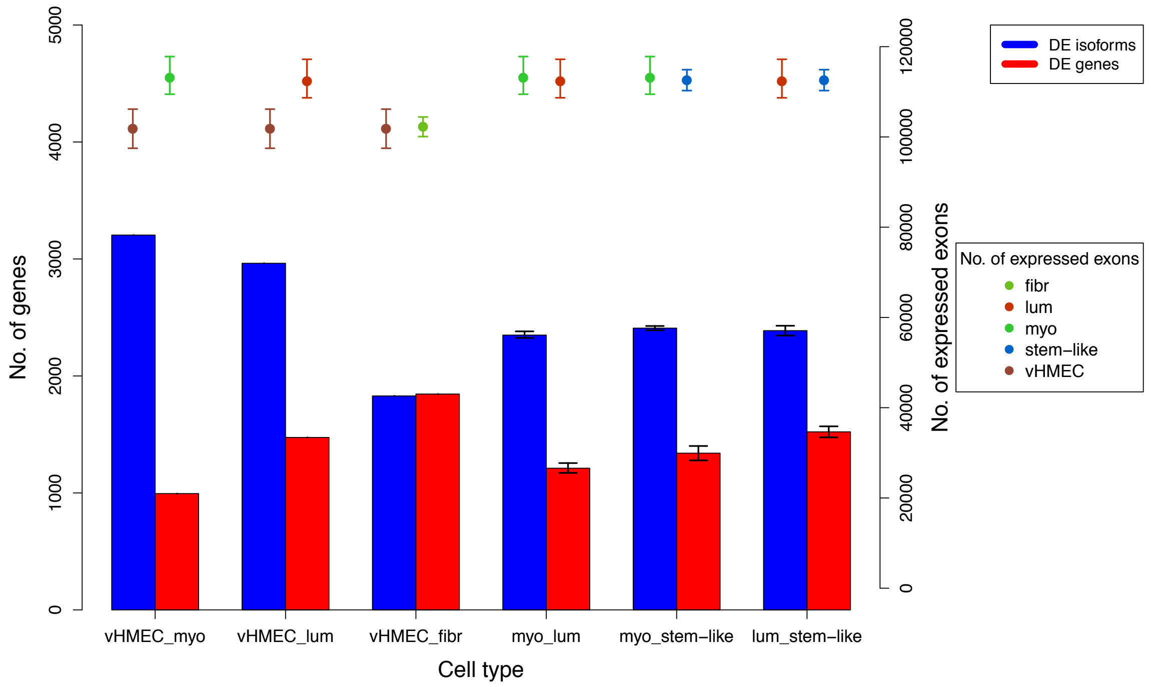

Supplementary Figure 2. Comparison of isoform genes and DE genes. Bar chart of No. of isoform genes (blue) and No. of differentially expressed genes (red) between different cell types within the same individuals with No. of expressed exons (dots) on the secondary y-axis. Error bar represents the minimum and maximum number across all individuals.

a

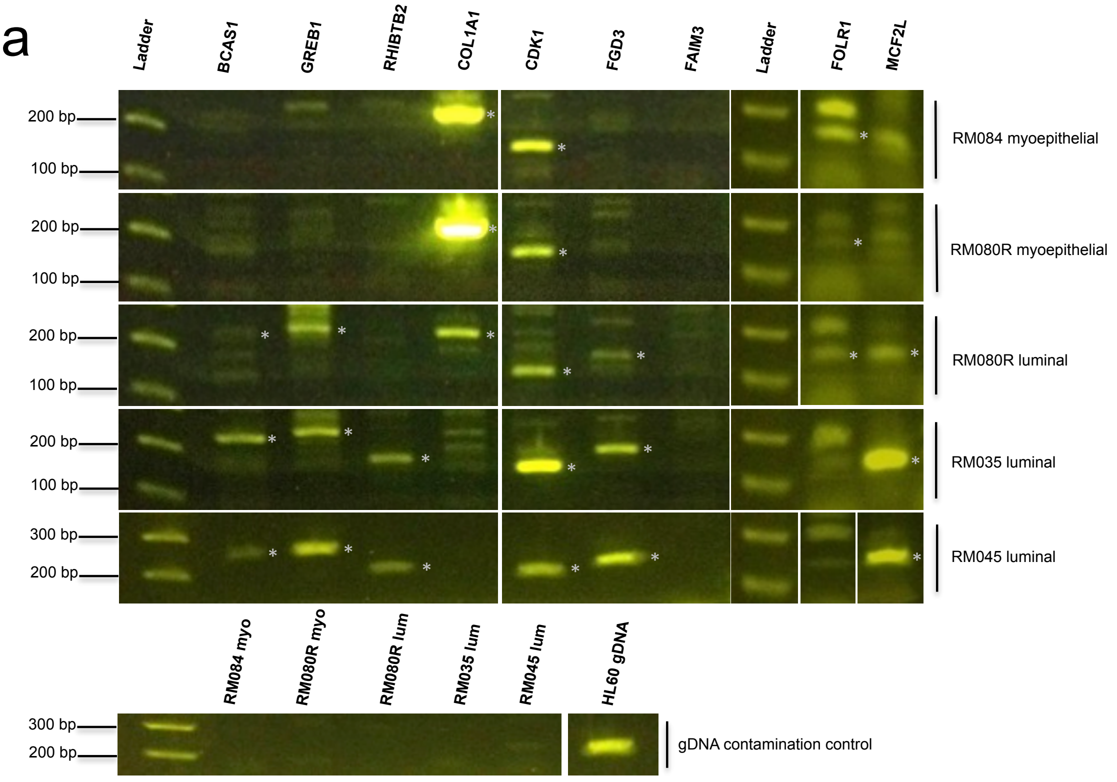

b

Sample	Gene Target								
	BCAS1	GREB1	RHBTB2	COL1A1	CDK1	FGD3	FAIM3	FOLR1	MCF2L
RM084 myoepithelial	Not present	Not present	Not present	Present	Present	Not present	Not present	Present	Non-specific
RM080R myoepithelial	Not present	Not present	Not present	Present	Present	Not present	Not present	Present	Non-specific
RM080R luminal	Present	Present	Not present	Present	Present	Present	Not present	Present	Present
RM035 luminal	Present	Present	Present	Not present	Present	Present	Not present	Non-specific	Present
RM045 luminal	Present	Present	Present	Not present	Present	Present	Not present	Not present	Present
Expected / No. of trials	5/5	5/5	4/5	4/5	3/5	5/5	5/5	4/4	3/3
**Expected / Total No. of trials**		**38/42 = 90.5%**							

Supplementary Figure 3. a. PCR was performed using primers flanking specific specific exon junctions to confirm their presence in RNA extracted from RM080R, RM035 and RM045 luminal samples and RM084 and RM080R myoepithelial samples. The expected size for the amplicons are (marked with *): BCAS1 (235 bp); GREB1 (247 bp); RHOBTB2 (187 bp); COL1A1 (244 bp); CDK1 (169 bp); FGD3 (201

bp); FAIM3 (198 bp); FOLR1 (153 bp) MCF2L (161 bp). To control for gDNA contamination in the samples, PCR was performed using primers against a non-transcribed region of the genome for each template and HL60 gDNA (positive control). **b.** The validation status of exon junctions in myoepithelial and luminal samples. Validation was judged by confirming the presence, absence or non-specific amplification of an amplicon containing the junction under investigation. All events were validated in at least one individual except for CDK1, which has a very low expression value (gene RPKM < 0.1).

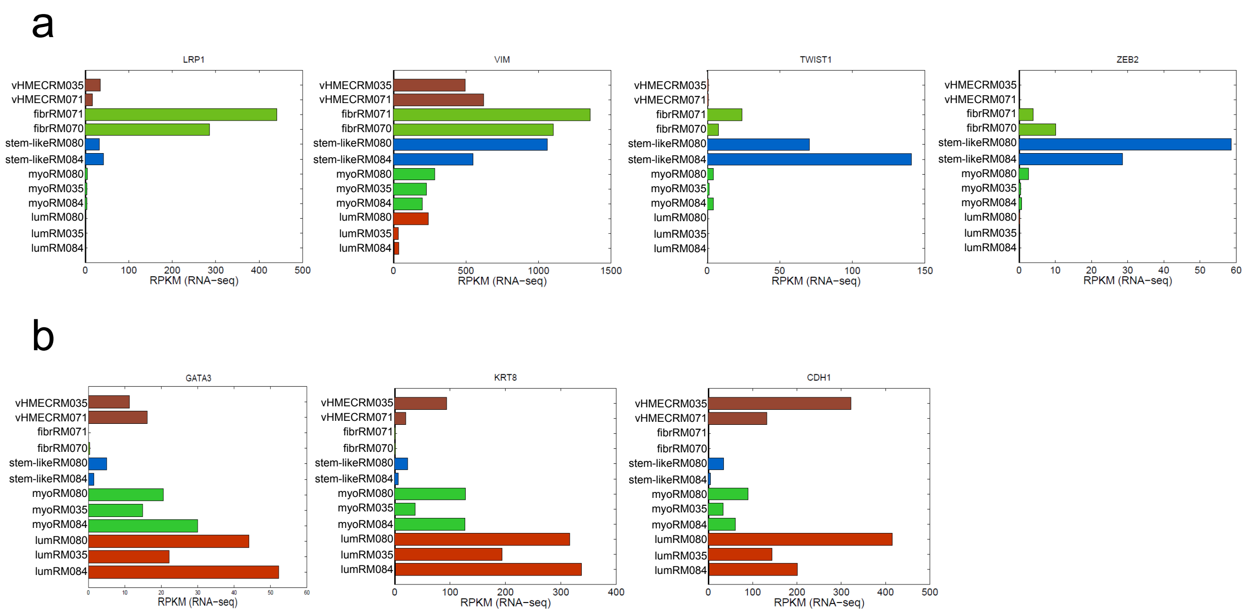

Supplementary Figure 4. Gene RPKM for progenitor/stem cell-specific genes (**a**) and luminal epithelial cell-specific genes (**b**).

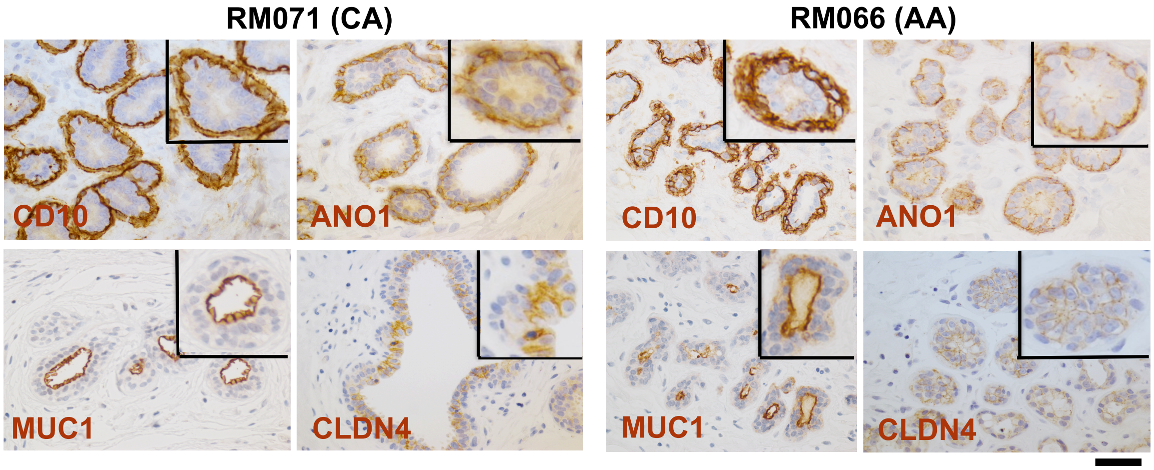

Supplementary Figure 5. Validation of myoepithelial and luminal-specific expression of selected markers by immunohistochemistry. Transcriptome analyses led to the identification of gene products with predicted myoepithelial or luminal cell-specific expression. These predictions were validated by immunostaining of genetically matched breast tissue sections as described in the Methods section. Shown are: upper row: expression pattern of CD10, the marker used to isolate myoepithelial cells, in a breast tissue section from African American donor RM066 (left panel) and validated myoepithelial-specific expression of anoctamin 1 (ANO1/TMEM16A) in Caucasian tissue donor RM071 (middle panel) and African American donor RM066 (right panel); lower row: expression pattern of MUC1, the marker used to isolate luminal cells, in a breast tissue section from Caucasian (CA) donor RM071 (left panel) and validated luminal cell-specific expression of claudin 4 (CLDN4) in Caucasian tissue donor RM084 (middle panel) and African American (AA) donor RM080 (right panel). Scale bar: 50µm**.**

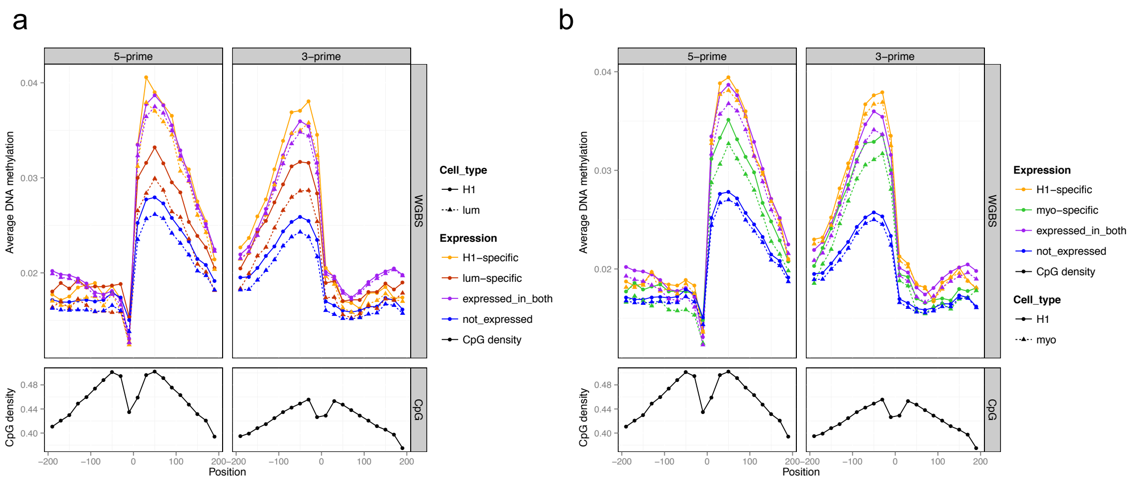

Supplementary Figure 6. WGBS profile at exon boundaries for hESC (H1) and luminal (**a**) or myoepithelial (**b**) isoforms. Average No. of CpGs (black, bottom panel) and average DNA methylation level profile at exon 5' and 3' +/- 200bp with 20bp bins in H1 (solid line with round dots) and luminal RM066 (**a**; dashed line with triangles) or myoepithelial RM045 (**b**; dashed line with triangles) WGBS libraries. Exons are divided into four groups according to isoform analysis in H1 vs RM084: exons expressed in both cell types (exon RPKM > 0.1 in luminal or myoepithelial and H1, purple), H1-specific exons (isoform exons expressed in H1 but not in luminal or myoepithelial, orange), luminal-specific exons (isoform exons expressed in luminal or myoepithelial but not in H1, red), and exons not expressed in either cell types (all other exons, blue). Maximum DNA methylation levels (peak) are calculated for each exon and t-tests are done to compare different exon groups. We observed a significant difference between not expressed exons and expressed exons, p-value < 10^{-10}, also between luminal-specific or myoepithelial-specific exons and exons expressed in H1, p-value < 10^{-4}.

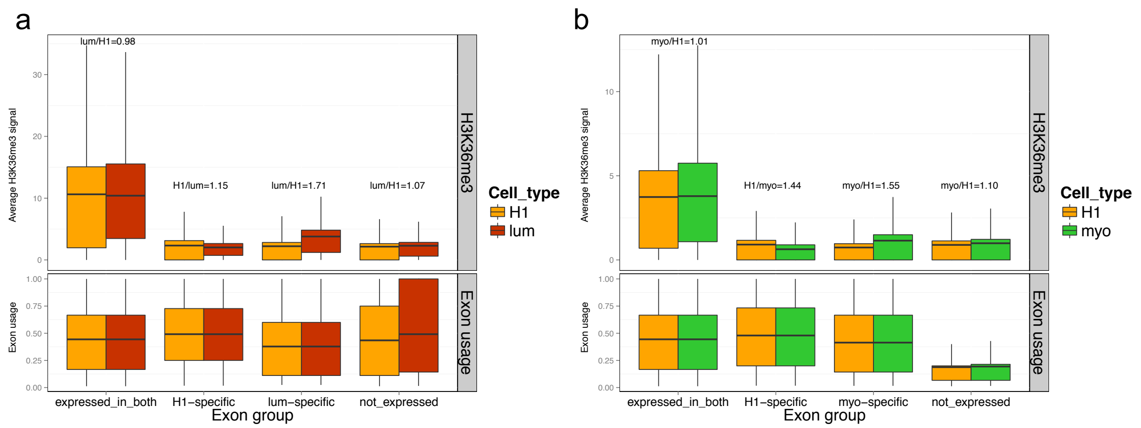

Supplementary Figure 7. H3K36me3 signal in exon bodies of expressed genes for hESC (H1) and luminal (**a**) or myoepithelial (**b**) isoforms. Average H3K36me3 signal levels for exons in expressed genes (gene RPKM > 0.1) in H1 (orange) and luminal RM080 (**a**; red) or myoepithelial RM080 (**b**; green). Exons are broken down into four groups according to isoform analysis in H1 vs RM084: exons expressed in both cell types (exon RPKM > 0.1 in H1 and luminal), H1-specific exons (isoform exons expressed in H1 but not in luminal or myoepithelial), luminal-specific exons (isoform exons expressed in luminal or myoepithelial but not in H1), and exons not expressed in either cell types (all other exons). Fold enrichment for average H3K36me3 signal levels are calculated for H1 vs luminal or myoepithelial in H1-specific exons, and for luminal or myoepithelial vs H1 in the other groups. Cell-type specific exons show increase in H3K36me3 signal level in corresponding cell types (e.g. H1-specific exon in). Exon usage are calculated for each exon with No. of transcripts containing this exon divided by total No. of transcripts of the gene according to Ensembl v65 ENST IDs.

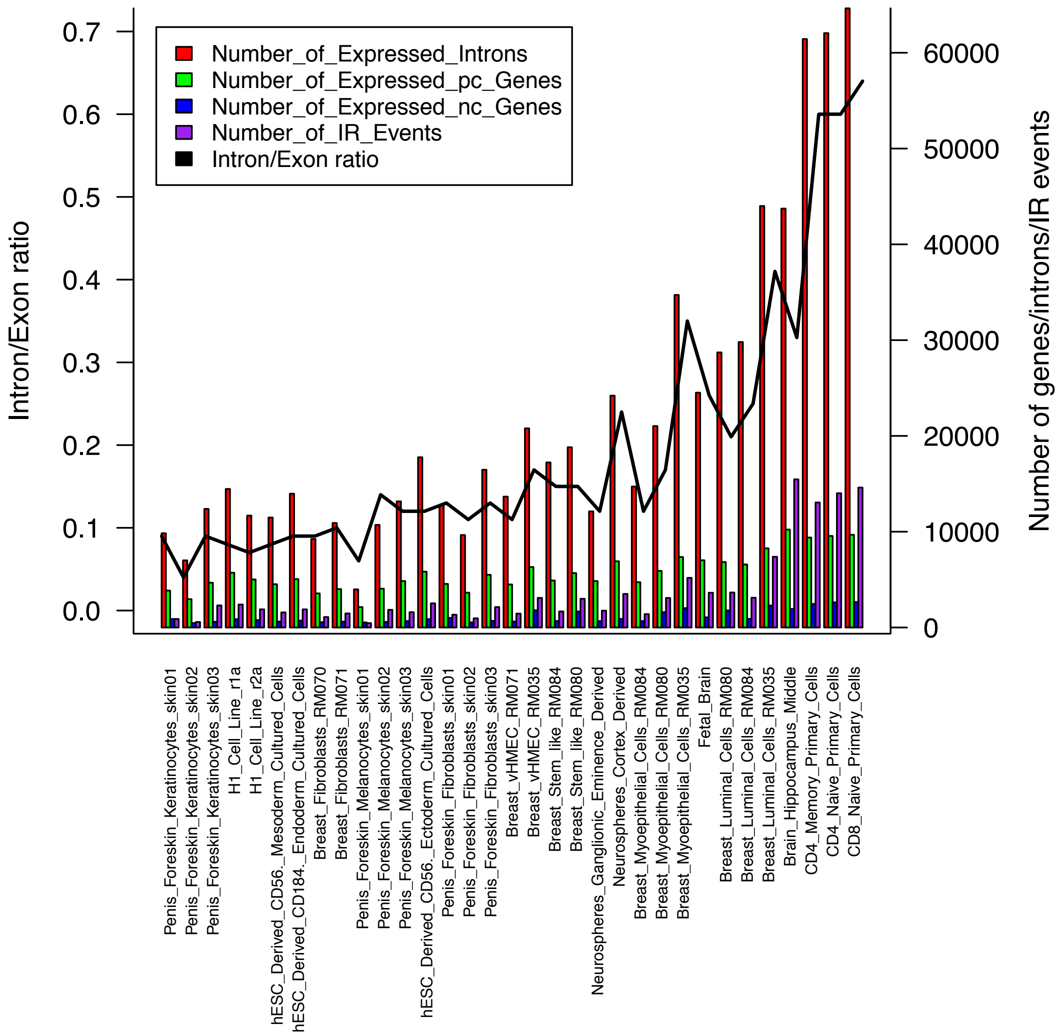

Supplementary Figure 8. Enumeration of expressed protein coding (pc) and non-coding (nc) genes, expressed introns and intron/exon ratio from human skin keratinocytes (left) to T-cells (right). Cell/tissue types are ordered by mean intron/exon ratio across replicates. All cell/tissue types datasets are from Roadmap consortium. Expressed is defined with RPKM > 1. Introns that overlap exons on the opposite strand have been excluded from the intron retention analysis of non

strand-specific as well as strand-specific libraries to make numbers shown on the plot consistent.

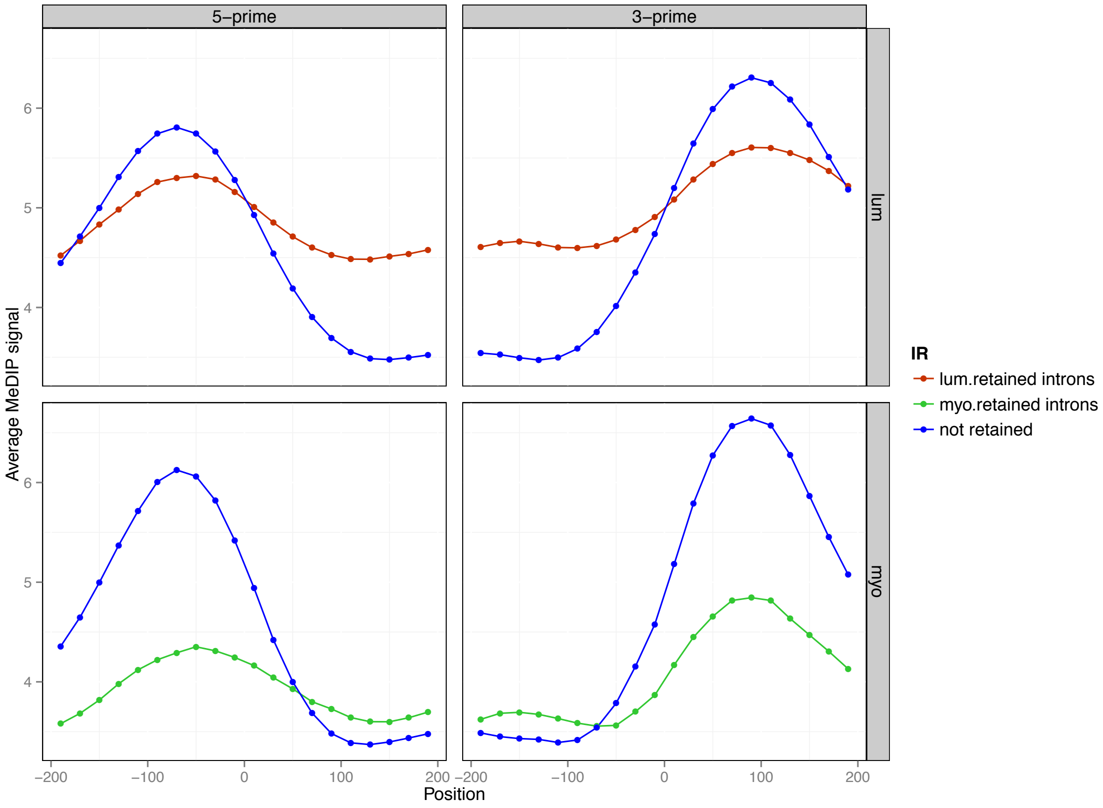

Supplementary Figure 9. MeDIP profile at intron boundaries. Average DNA methylation level profile at intron 5' and 3' +/- 200bp with 20bp bins in luminal RM035 (top panel) and myoepithelial RM035 (bottom panel) MeDIP libraries. Introns are divided into retained introns (red in luminal, and green in myoepithelial), and not retained introns (blue) according to intron retention analysis in RM084.

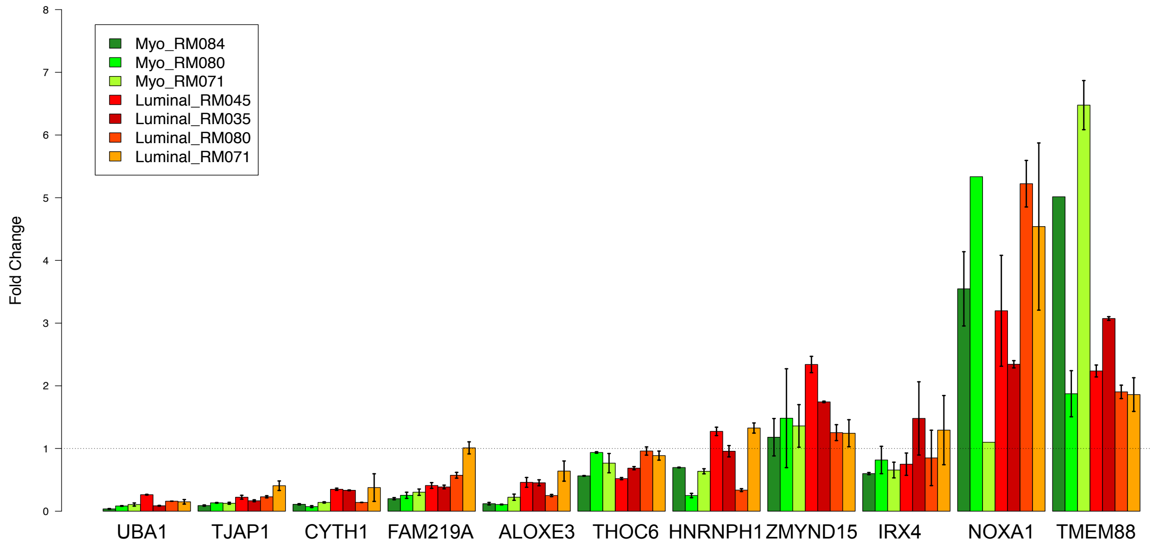

Supplementary Figure 10. RT-qPCR showing the fold change of intron-retained transcripts expression compared to correctly spliced transcripts expression across 3 myoepithelial (green) and 4 luminal (red) samples for 11 different genes. Mean and standard deviation was computed for every triplicate experiment.

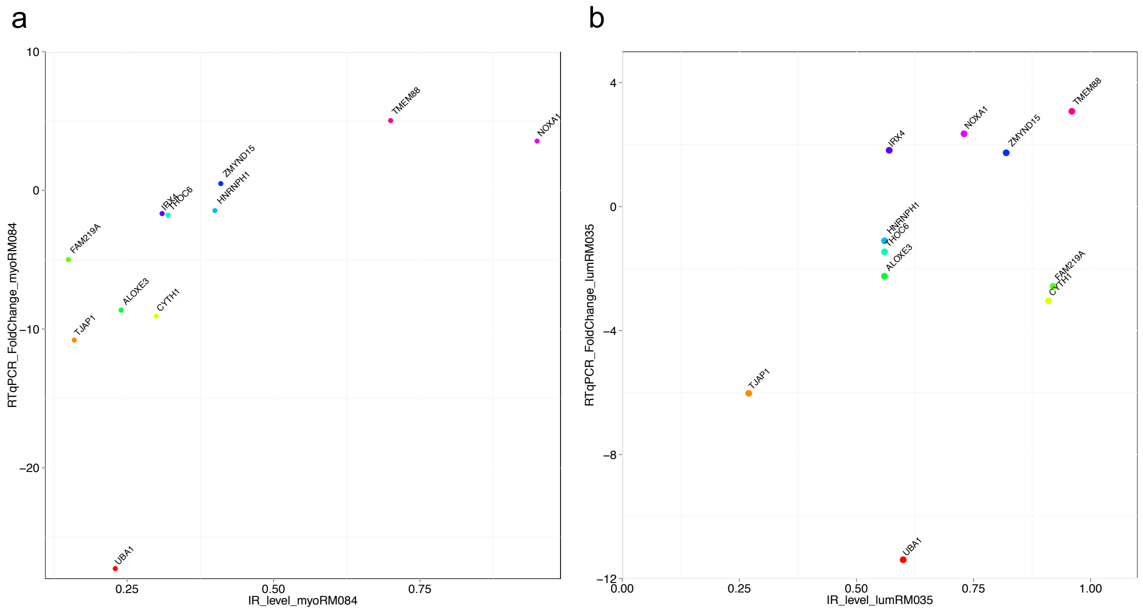

Supplementary Figure 11. 2D plots showing intron retention level (x-axis) and RT-qPCR fold change (y-axis) across 11 genes for RM084 myoepithelial (**a**) and RM035 luminal (**b**).

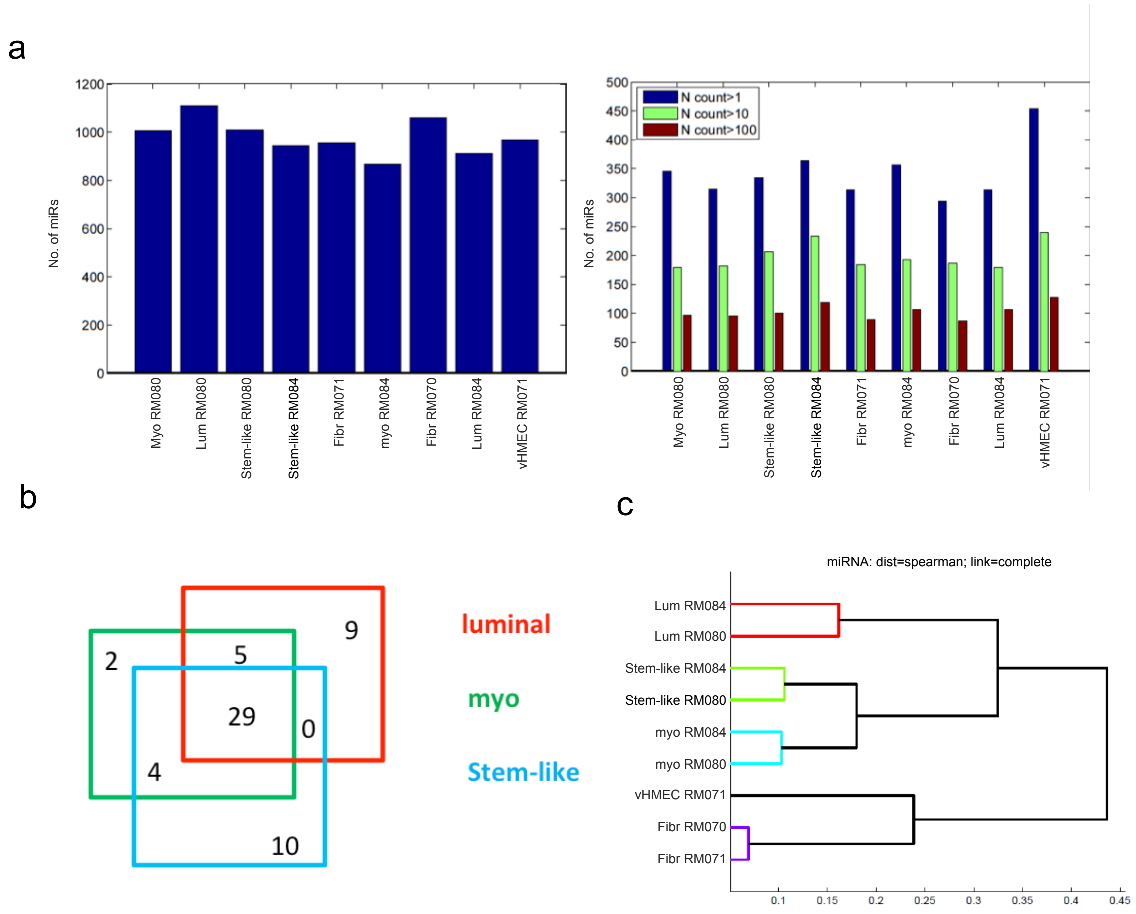

Supplementary Figure 12. Expression of miRNAs in mammary gland cell types**. a.** Unique miRNA species detected (>0.1 reads per million mapped; left) in mammary gland cell types and their abundance (right). **b.** Highly expressed miRNA common to all mammary cell types (see **Supplementary Data 7** for accessions and normalized abundance). **c.** Unsupervised clustering of miRNAs across breast cell-types.

a

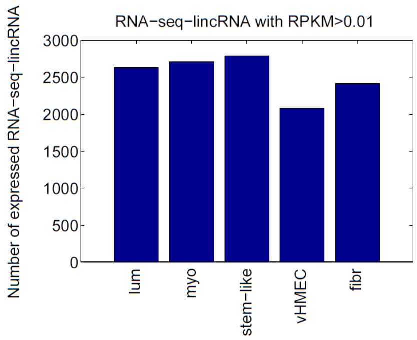

b

Min RPKM	Number of lincRNAs
0.01	1328
0.1	636
1	141
10	13
100	1

c

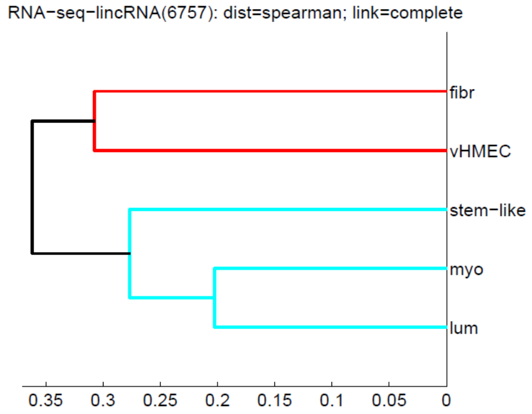

Supplementary Figure 13. Expression of lincRNAs in mammary gland cell types**.** **a.** Unique lincRNA species detected (>0.1 reads per million mapped) in breast cell types **b.** Unique lincRNAs common to all mammary cell types (see **Supplementary Data 8** for accession and normalized abundance). **c.** Unsupervised clustering of all lincRNAs across mammary cell types.

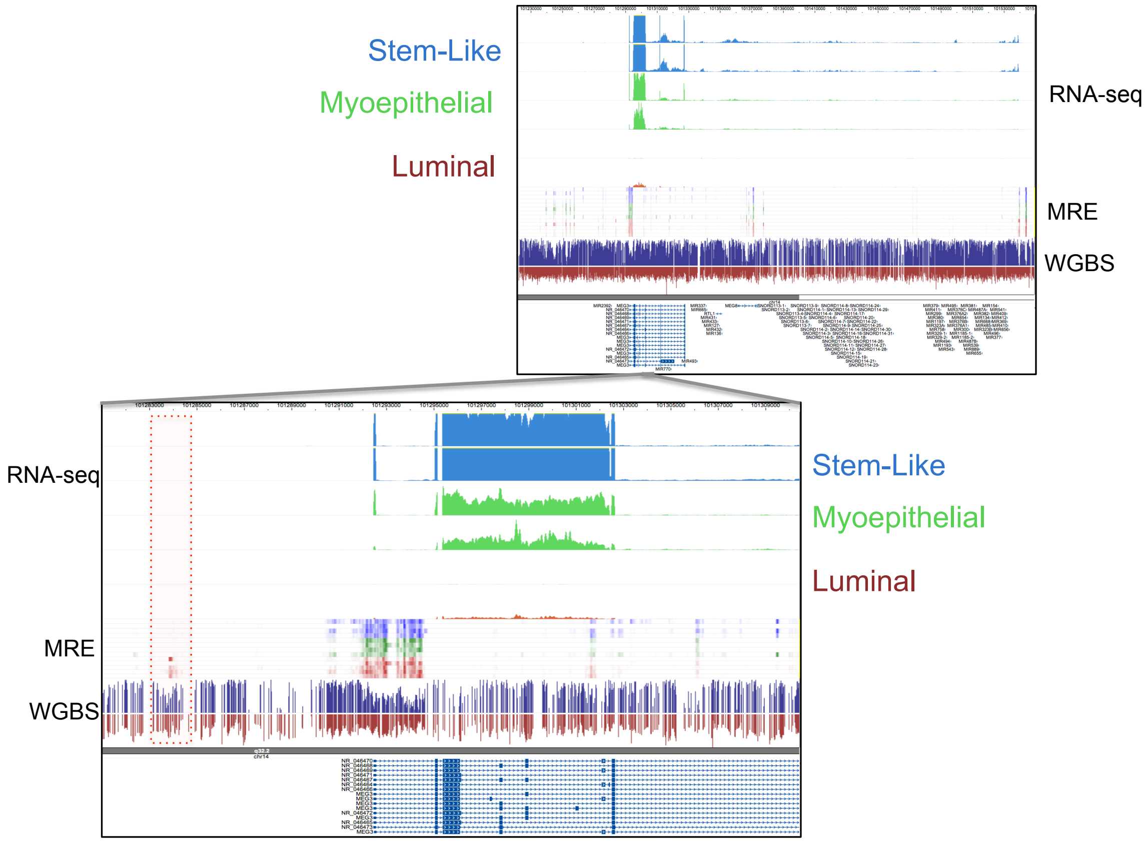

Supplementary Figure 14. The maternally imprinted lincRNA *MEG3* is specifically silenced in luminal cells. Genome browser screen shots showing RNA-seq, MRE-seq and WGBS datasets for stem-like (blue), myoepithelial (green) and luminal epithelial (red) mammary cell types.

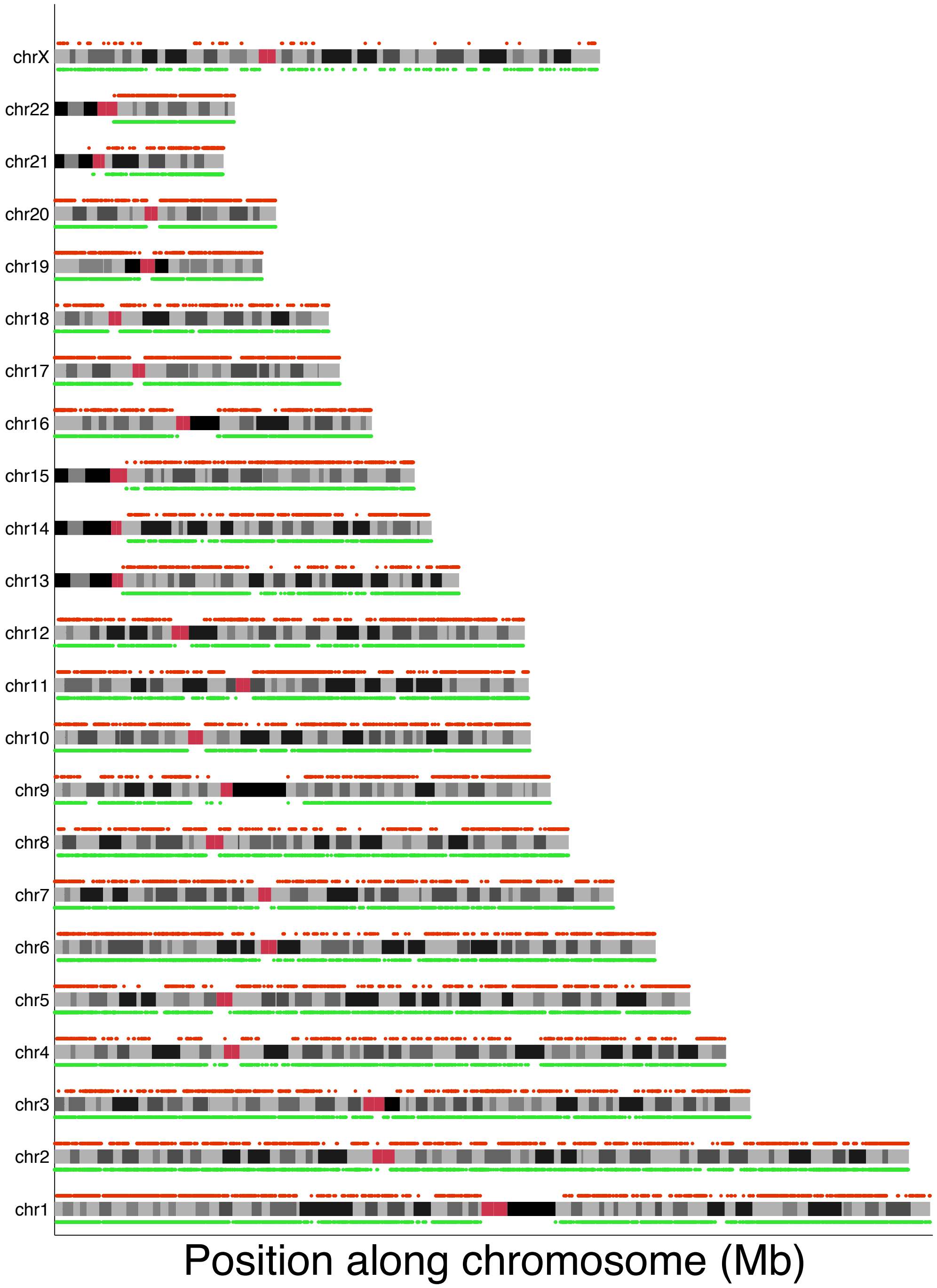

Supplementary Figure 15. Geographic distribution of luminal (red) and myoepithelial (green) UMRs along the chromosomes.

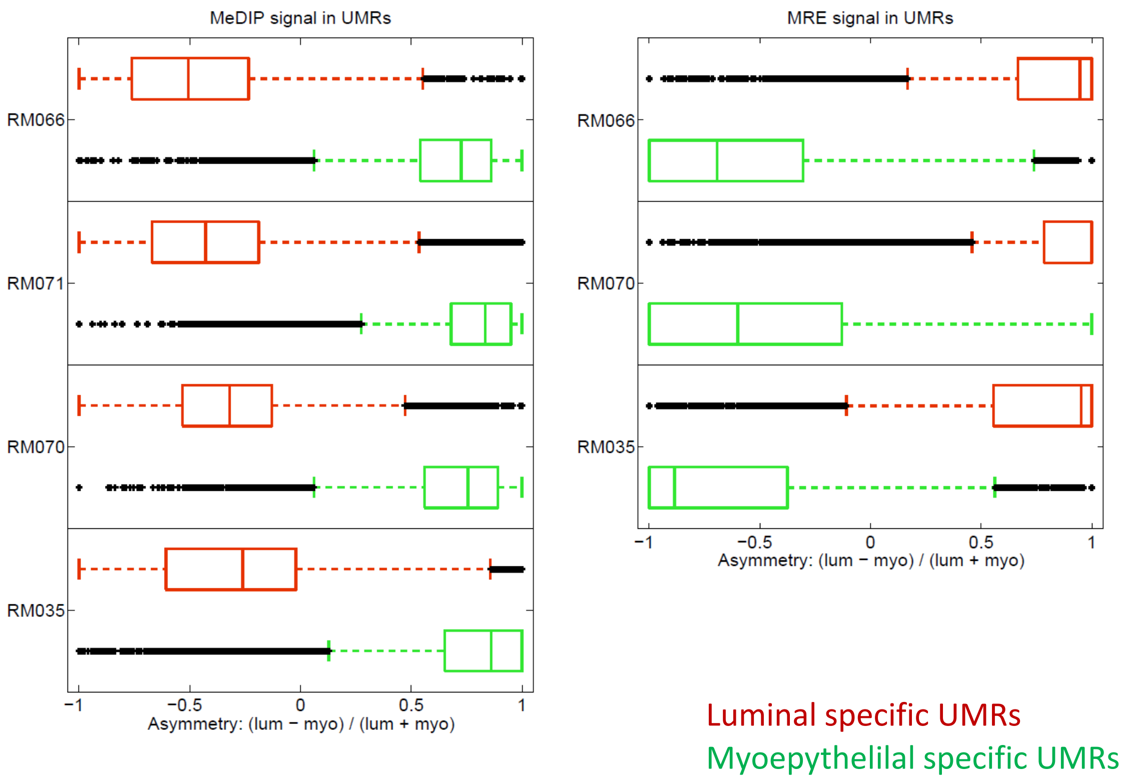

Supplementary Figure 16. For each UMR identified by WGBS we calculated the normalized MeDIP-seq (methylated, left panel) and MRE-seq (unmethylated, right panel) signal. From this we calculated methylation asymmetry between MeDIP-seg and MRE-seq signals in luminal and myoepithelial cells defined as (signal(lum)-signal(myo))/(signal(lum)+signal(myo)). For each donor (identified by RM#) the box plot shows a distribution of asymmetry in luminal specific UMRs (red) and myoepithelial specific UMRs (green). As expected MeDIP-seq confirms low methylation in luminal cells (asymmetry <0) in luminal specific UMRs and high methylation in luminal (asymmetry >0) in myoepithelial specific UMRs. Conversely, for MRE-seq data that detects unmethylated CpG locations, the opposite is true.

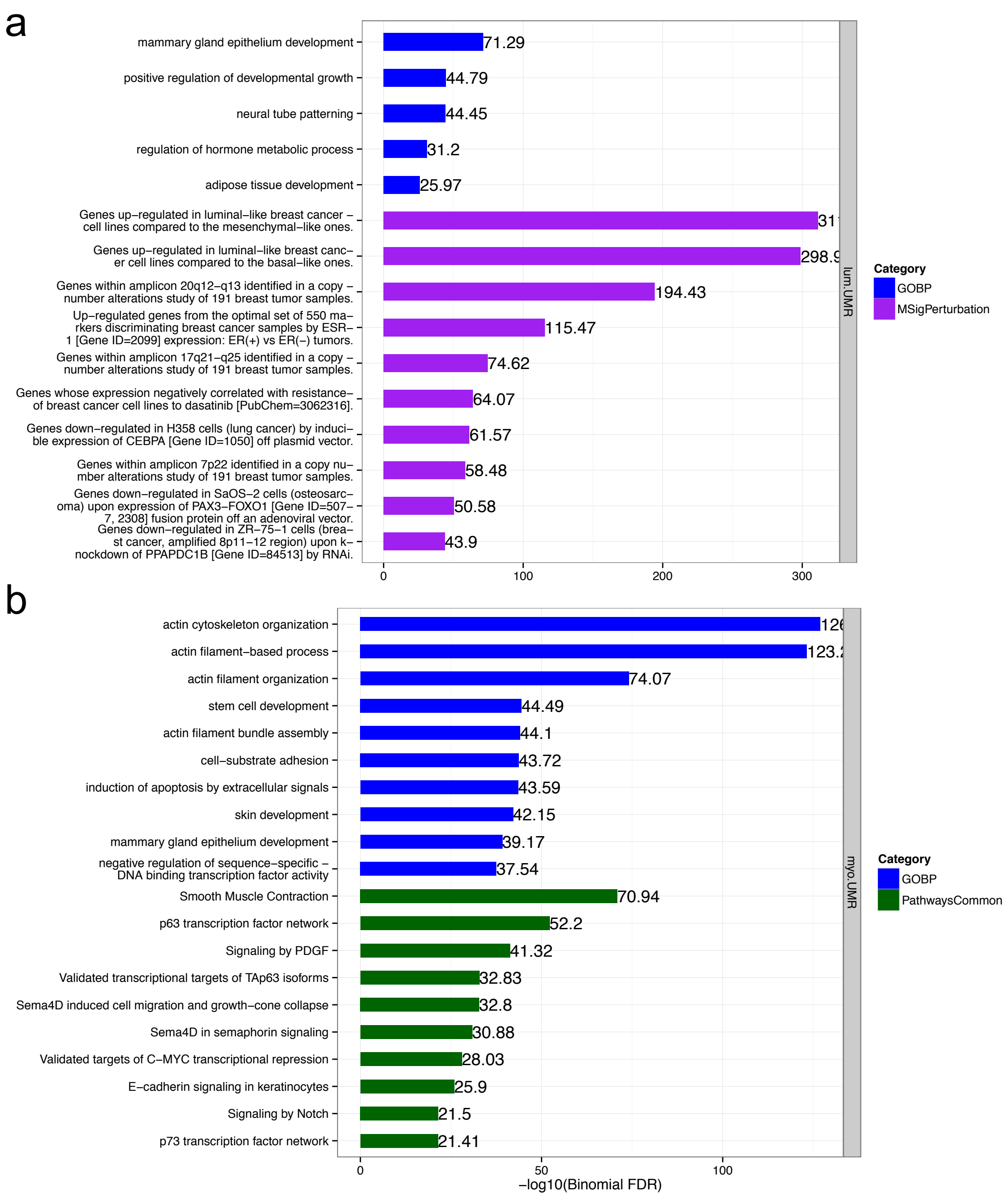

Supplementary Figure 17. Top 10 GREAT (PMID 20436461) functional enriched (binomial and hypergeometric FDR < 0.05) terms in GO biological process (GOBP), Molecular Signature Database Perturbation (MSigPerturbation), and Pathways

Common for UMRs with at least 5 CpGs in **a.** luminal (32947 regions), and **b.** myoepithelial (19213 regions).

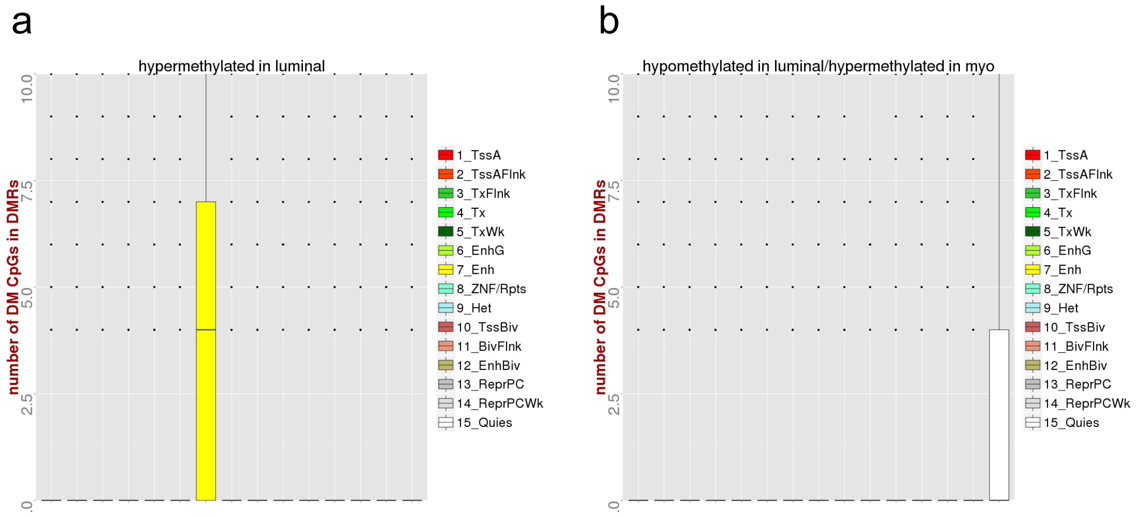

Supplementary Figure 18. Overlap of myoepithelial chromatin states with UMRs from myoepithelial and luminal cell types. **a.** Overlap with myoepithelial UMRs. **b.** Overlap with luminal UMRs.

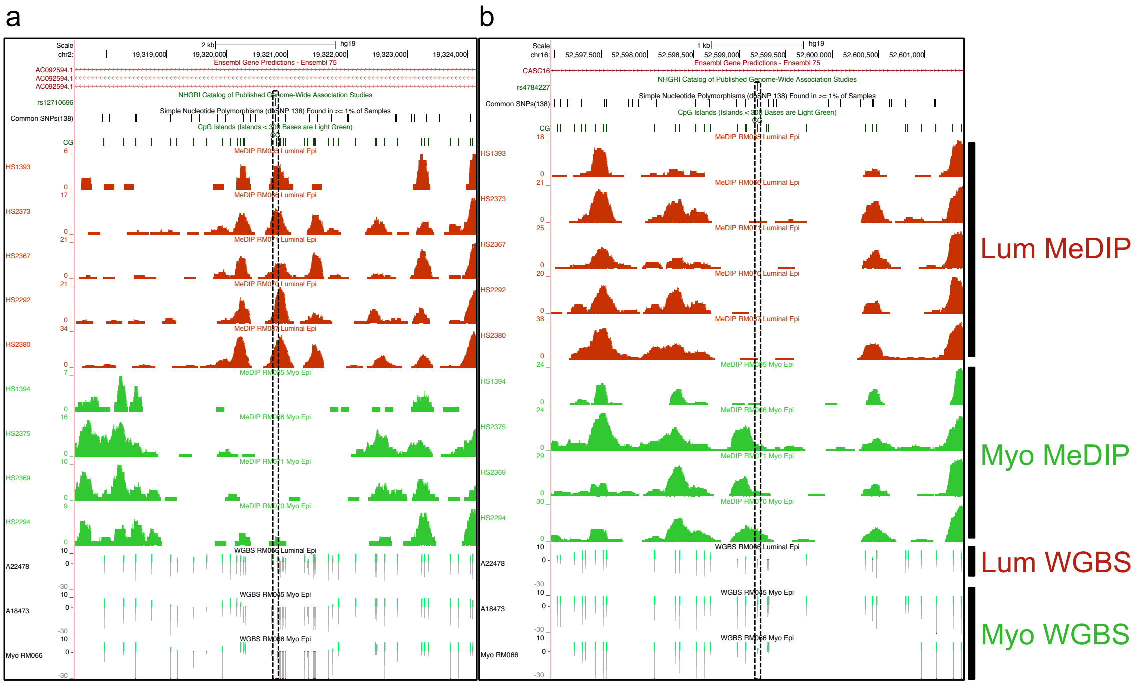

Supplementary Figure 19. UCSC view of the methylation data (MeDIP) and WGBS for luminal and myo epithelial cells showing identifies myo (**a**) and luminal (**b**) specific UMR overlapping GWAS SNPs linked to breast cancer risk.

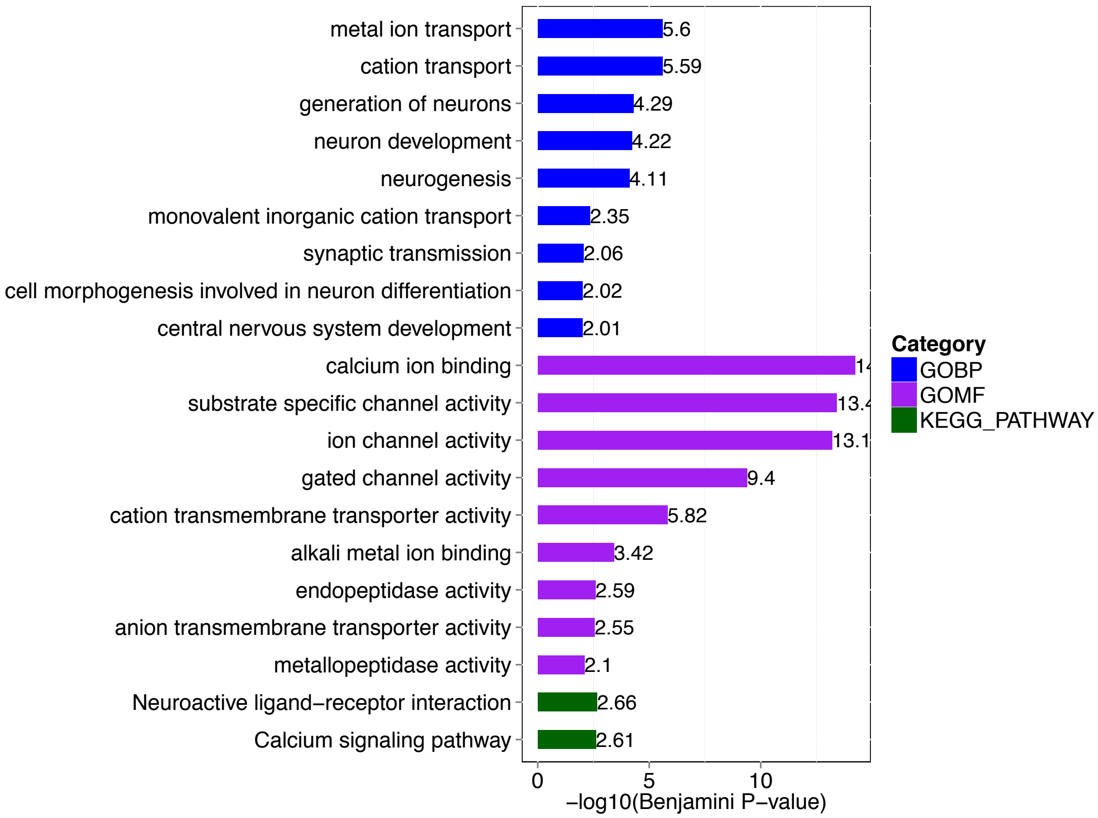

Supplementary Figure 20. DAVID functional enriched terms (Benjamini corrected p-value < 0.01) in GO biological process (GOBP), GO molecular function (GOMF), and KEGG pathway for luminal and myoepithelial isoforms shared by RM035, RM080 and RM084.

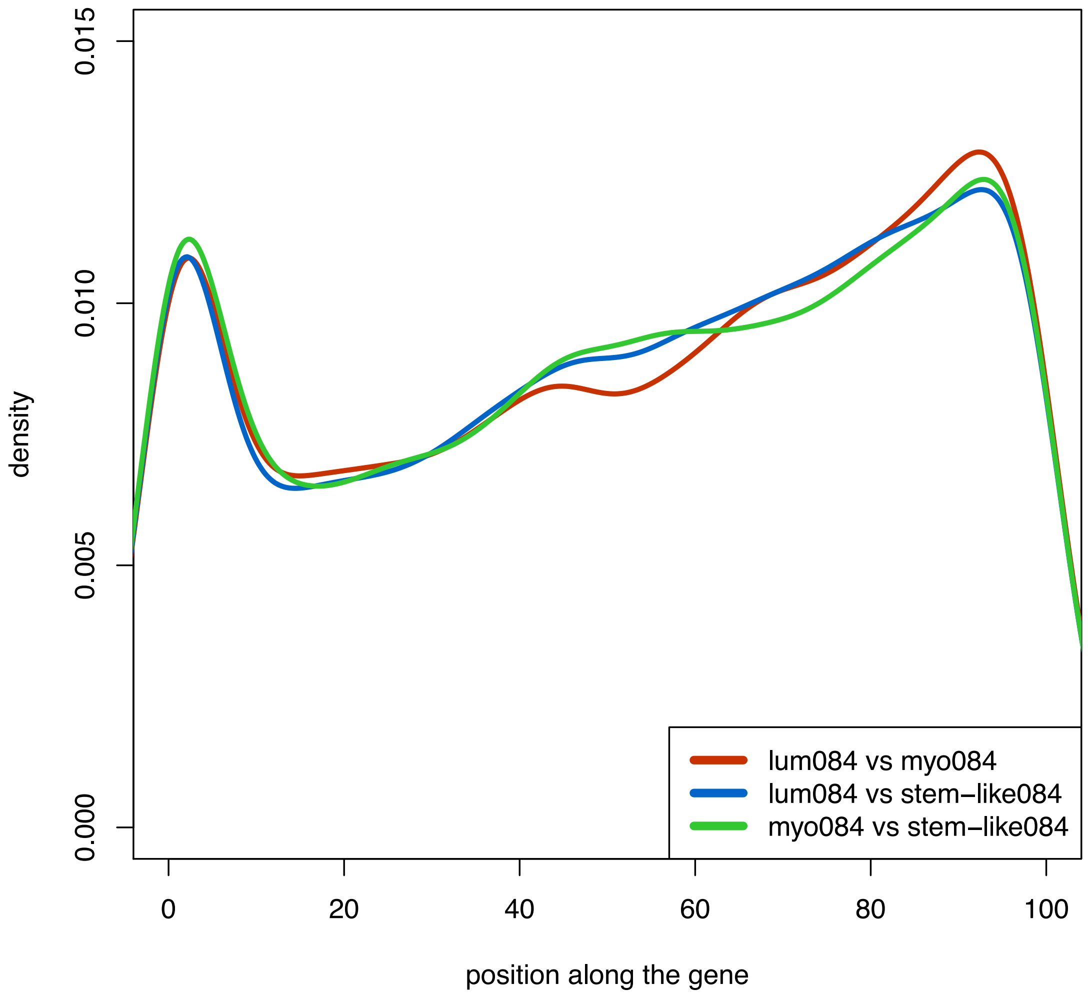

Supplementary Figure 21. Cassette exons position along the length of the gene (normalized to 0-100) in RM084 luminal vs myoepithelial (red), luminal vs stem-like (blue), and myoepithelial vs stem-like (green). Cassette exons are enriched at two ends of the gene.

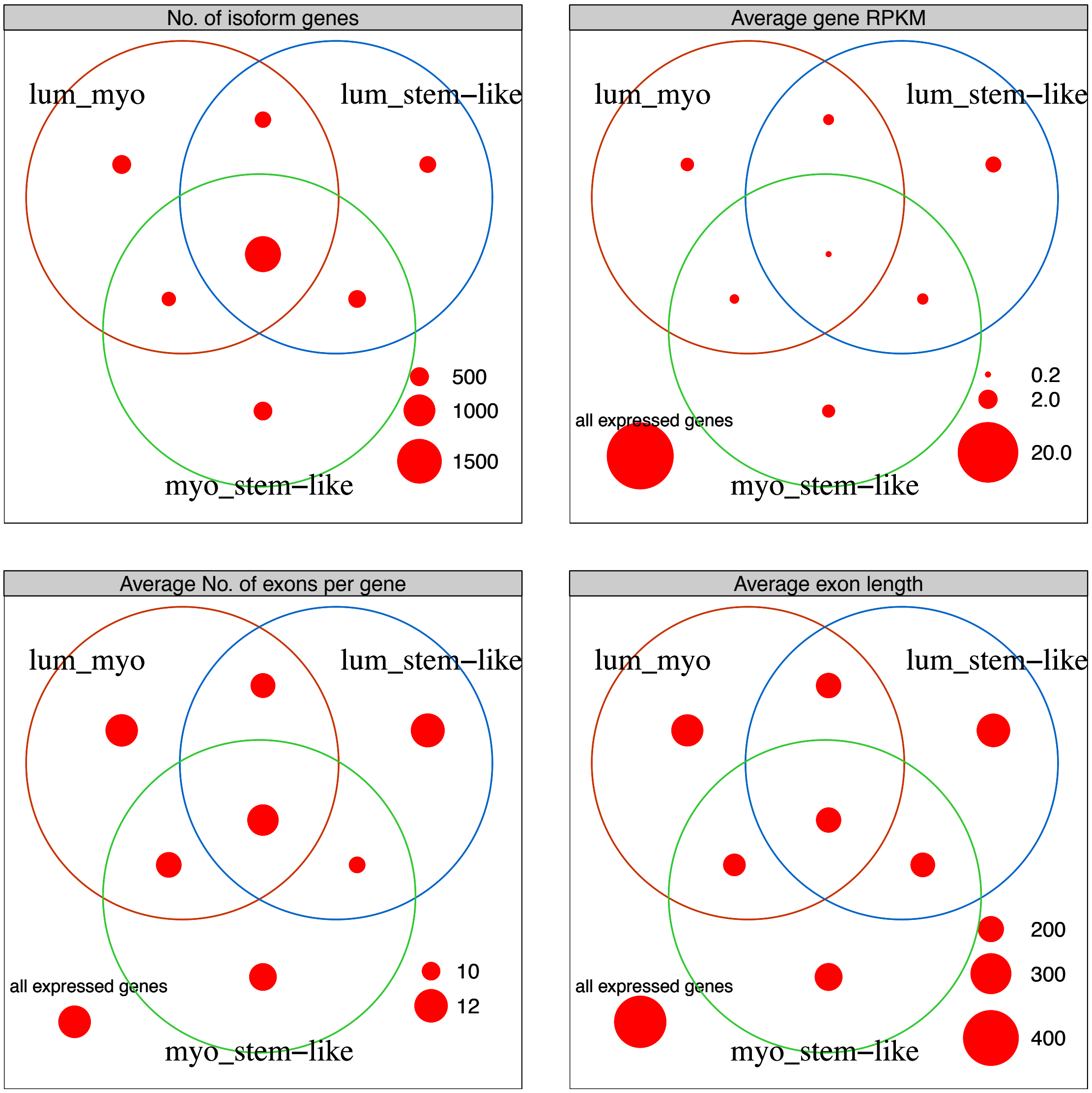

Supplementary Figure 22. RM084 isoform Venn diagrams. Venn diagrams of RM084 isoform genes between luminal and myoepithelial (red), luminal and stem-like (blue), and myoepithelial and stem-like (green) with bubble plots representing No .of isoform genes, average gene expression level, average No. of exons per gene, and average isoform exon length respectively. Isoforms between different cell types show significant overlap. The expression level of isoform genes are much lower than

average expressed genes, but average No. of exons per gene and exon length are similar to all expressed genes.
